# Triterpenes as Potential Drug Candidates for Rheumatoid Arthritis Treatment

**DOI:** 10.3390/life13071514

**Published:** 2023-07-05

**Authors:** Célia Faustino, Lídia Pinheiro, Noélia Duarte

**Affiliations:** iMed.ULisboa, Research Institute for Medicines, Faculdade de Farmácia, Universidade de Lisboa, Avenida Prof. Gama Pinto, 1649-003 Lisbon, Portugal; cfaustino@ff.ulisboa.pt

**Keywords:** rheumatoid arthritis, inflammation, triterpenes, celastrol, betulinic acid, ginsenosides, saponins

## Abstract

Rheumatoid arthritis (RA) is a chronic autoimmune inflammatory disease characterized by joint inflammation, swelling and pain. Although RA mainly affects the joints, the disease can also have systemic implications. The presence of autoantibodies, such as anti-cyclic citrullinated peptide antibodies and rheumatoid factors, is a hallmark of the disease. RA is a significant cause of disability worldwide associated with advancing age, genetic predisposition, infectious agents, obesity and smoking, among other risk factors. Currently, RA treatment depends on anti-inflammatory and disease-modifying anti-rheumatic drugs intended to reduce joint inflammation and chronic pain, preventing or slowing down joint damage and disease progression. However, these drugs are associated with severe side effects upon long-term use, including immunosuppression and development of opportunistic infections. Natural products, namely triterpenes with anti-inflammatory properties, have shown relevant anti-arthritic activity in several animal models of RA without undesirable side effects. Therefore, this review covers the recent studies (2017–2022) on triterpenes as safe and promising drug candidates for the treatment of RA. These bioactive compounds were able to produce a reduction in several RA activity indices and immunological markers. Celastrol, betulinic acid, nimbolide and some ginsenosides stand out as the most relevant drug candidates for RA treatment.

## 1. Introduction

Nature has always been the foundation for the discovery of folk medical treatments and many drugs used in modern medicine. Currently, the use of natural products and natural supplements is progressively increasing, and their scientific validation is a priority to guarantee the safe use of these products. In addition, natural products derived from plants, marine organisms and microorganisms, as well as their synthetic derivatives designed based on their distinctive pharmacophores, play a pivotal role in the process of drug discovery and development. This contribution is reflected by the significant number of drug molecules recently introduced to the market, as extensively emphasized in various reviews [1,2,3,4]. Notably, a considerable 41% of small-molecule anti-cancer drugs approved between 1981 and 2019 possess structures derived from natural products (e.g., paclitaxel, vincristine and etoposide). The impact of natural products extends beyond cancer therapeutics and encompasses other therapeutic areas such as cardiovascular diseases (e.g., statins, digoxin and warfarin), multiple sclerosis (e.g., fingolimod), protozoal infections (e.g., quinine and artemisinin) and a plethora of other infectious diseases [1,5].

Earth’s biodiversity is still far from being fully explored in terms of discovering new bioactive compounds. The structural diversity of secondary metabolites is a rich biogenetic supply for the discovery of novel drugs when compared to synthetic molecules, offering hit and lead compounds for rational drug design [6]. Among the diverse families of natural products (e.g., terpenoids, steroids, phenolic compounds and alkaloids), triterpenes are an important group of phytochemicals, possessing a wide array of biological effects, which have been extensively documented in the scientific literature [7,8,9,10,11]. Among these effects, the anti-microbial [12,13], anti-tumor [14,15,16], anti-diabetic [10], anti-cholesterol [17], anti-inflammatory [18] and immunomodulatory [19] activities have gathered considerable attention within the pharmaceutical area [20,21]. Particularly, numerous scientific studies have highlighted the potent anti-inflammatory properties of triterpenes, making them potentially relevant in the treatment of inflammatory conditions such as arthritis and related diseases [22].

Arthritis is an acute or chronic joint disease usually associated with joint stiffness, pain, inflammation, swelling and decreased range of motion [23,24]. There are more than 100 different types of arthritis, the most common being non-inflammatory degenerative arthritis known as osteoarthritis [23,24]. Rheumatoid arthritis (RA) is the most common autoimmune inflammatory type of arthritis. Inflammatory arthritis can also be caused by other factors, such as crystal deposition-induced inflammation (e.g., gout, pseudogout) or infections (e.g., septic arthritis). Inflammatory arthritis has also been associated with other autoimmune connective tissue diseases (e.g., systemic lupus erythematosus) and extra-articular comorbidities [23,24].

RA is an important cause of disability and its prevalence varies globally (Table 1), with higher rates in industrialized countries, which could possibly be explained by the higher exposures to environmental factors. Nevertheless, other risk factors are also considered in the development of RA, such as advancing age, female sex, smoking and stress, among others (Table 2). RA most commonly affects the joints, but it is also considered a systemic disease because it can also affect other organs, such as the cardiovascular or respiratory system (Table 2) [23]. This chronic autoimmune condition represents a substantial health, social and economic burden, resulting in chronic pain and disability, impacting work performance and interfering with daily tasks, decreasing the patient’s quality of life and contributing to anxiety and depression [23]. 

Currently, RA treatment is based on anti-inflammatory drugs and disease-modifying anti-rheumatic drugs, aiming at reducing joint inflammation and pain, protecting joints and other tissues from permanent damage and slowing the progression of RA. The sustained use of these drugs is associated with severe side effects such as stomach upset, heartburn, internal bleeding, osteoporosis, adrenal suppression or development of opportunistic infections. Furthermore, some drugs are very expensive and non-effective in a percentage of RA patients [26,27]. Therefore, the discovery of new drugs with fewer side effects is essential and should embrace several approaches, including the study of natural products and/or their synthetic derivatives. In recent years, several reviews have reported the anti-RA effects of natural compounds and herbal drugs [28,29,30,31,32]. However, the information is scattered amongst the diverse compound families and plant sources. As far as we know, a comprehensive review gathering the most recent studies on triterpenoids with RA-related effects is still missing. This review covers and discusses the latest results on triterpenes, natural products with anti-inflammatory properties, which have been shown to be effective against RA both *in vitro* and *in vivo* in several animal models. For a better comprehension several aspects of RA will firstly be addressed, including the etiology, pathogenesis, current treatment and a summary of the different animal models used in the *in vivo* studies.

## 2. Materials and Methods

The literature search was carried out during January 2023 using PubMed, Web of Science and ScienceDirect, and an appropriate combination of keywords and truncations adapted for each database was used (for example, combinations of triterpenes with arthritis, rheumatoid arthritis, inflammation and treatment). Only peer-reviewed research articles in the English language and published in a six-year timespan (2017–2022) were considered. The studies were individually screened by the authors based on quality, accuracy and relevance to the aim of the review. Mendeley reference manager software (2020) was used to manage the references and eliminate duplicates.

## 3. Rheumatoid Arthritis

RA is a systemic autoimmune and chronic inflammatory disease that primarily affects the joints, causing inflammation and swelling of the synovium with subsequent destruction of articular structures, pain and disability [24,33]. Typically, RA symmetrically affects small peripheral joints (hands, wrists and feet) but may progress to involve proximal joints if not treated [24,33]. The acute-phase response to inflammation is signaled by raised serum levels of C-reactive protein and increased erythrocyte sedimentation rate, which are relevant disease assessment biomarkers. Systemic inflammation associated with RA is responsible for extra-articular comorbidities, including cardiovascular disease, lymphoma, interstitial lung disease, pulmonary fibrosis, vasculitis, metabolic syndrome, type 2 diabetes, atherosclerosis, osteoporosis, anemia, dry keratoconjunctivitis and depression, resulting in increased morbidity and mortality in RA patients [24,33,34].

The presence of autoantibodies against post-translational modified proteins, namely anti-citrullinated protein antibodies (ACPAs), usually measured as anti-cyclic citrullinated peptide antibodies, is a hallmark of the disease, along with less specific autoantibodies that bind the Fc region of immunoglobulin G (IgG), known as rheumatoid factors (RFs), of various isotypes (e.g., IgM, IgG and IgA) [33,35,36]. These antibodies can be found in 50–70% of RA patients [33,37] and are currently used as biomarkers for diagnostic purposes. Based on the presence or absence of these antibodies in serum, RA can be subdivided in seropositive or seronegative, respectively [33]. Furthermore, RF is a predictive factor for occurrence of rheumatoid nodules, which are the most common extra-articular feature of RA [34]. The presence of this autoantibody has been detected in approximately 90% of RA patients with nodular disease [34]. Autoantibodies can already be detected decades before disease onset [37] and seropositivity is associated with a more aggressive RA phenotype and increased mortality [36,37].

### 3.1. Etiology

RA prevalence increases with population aging, peaking in the 60–64 and 65–69 age groups for women and men, respectively, according to 2019 data [38]. Women are 2–3 times more likely to develop RA than men (Table 1). Sex hormones may play a role in disease development since susceptibility to RA increases in post-menopausal women while breastfeeding has been associated with a decreased risk of developing RA [39].

Although the etiology of RA is still unknown, disease onset and progression are likely the result of an interplay between (epi)genetic and environmental factors and the presence or absence of autoantibodies. The heritability of RA is around 50% for seropositive RA and about 20% for seronegative RA [40]. Genetic predisposition for developing RA has been mainly associated with human leukocyte antigen (HLA) class II genotypes, namely HLA-DRB1 alleles of the major histocompatibility complex (MHC), which share a conserved amino acid sequence in their peptide-binding groove, known as the “shared epitope” [41,42]. Shared epitope-positive HLA-DRB1 alleles are associated with ACPA production and an increased risk of developing severe seropositive RA [36,41,42]. Several non-HLA-related genetic associations in RA have also been detected, such as polymorphisms in PTPN22, a shared autoimmunity gene also associated with systemic lupus erythematosus, type 1 diabetes mellitus, juvenile idiopathic arthritis and vasculitides involved in the regulation of both T cells and B cells, which is linked to an increased risk of severe seropositive RA, especially in Caucasians and Africans [43]. Similarly, single-nucleotide polymorphisms in the TNFAIP3 gene locus are related to both inflammatory and autoimmune diseases and have been associated with RA susceptibility [41]. TNFAIP3 encodes the (de)ubiquitinating enzyme A20 that inhibits tumor necrosis factor (TNF)-induced activation of nuclear factor kappa-B (NF-κB), and TNFAIP3 gene-deficient mice develop spontaneous arthritis [41]. Epigenetic factors are also relevant contributors to the disease pathogenesis, for instance, the unique DNA methylome pattern of RA fibroblast-like synoviocytes (FLSs) is different from that of osteoarthritic FLSs, and this persistent differential methylation contributes to the aggressive proliferative phenotype of RA FLSs [44].

Smoking, fine particulate matter exposure and periodontal disease are known environmental risk factors for developing RA [24,33,45]. Lung exposure to smoke, silica dust and other particulate air pollutants can induce the expression of calcium-dependent peptidyl-arginine deiminases (PADs), which convert arginine to citrulline, thus increasing protein citrullination and triggering ACPA production in genetically susceptible individuals [42,45,46]. Similarly, aberrant citrullination of endogenous peptides by Porphyromonas gingivalis PAD, a major causative agent of periodontitis, may be involved in breach of tolerance to citrullinated proteins in RA [47,48]. Other infectious agents, such as mycobacteria or Epstein–Barr virus, can trigger RA via molecular mimicry [33,42]. Recently, gut microbiome dysbiosis has been implicated in early RA [49], corroborating data from animal models of arthritis [50]. Furthermore, alterations in common oral, gastrointestinal and pulmonary microbial populations have been associated with ACPA status [51].

### 3.2. Pathogenesis

Both adaptative and innate immune systems are involved in the pathogenesis of RA. A pre-RA phase comprises early generation of ACPAs that bind citrullinated residues on many self-proteins, including collagen type II (CII), vimentin, α-enolase, fibronectin, fibrinogen and histones [35,36,42,47].

Mucosal surfaces, especially the lung, are potential trigger sites [35,36,47], consistent with mucosal microbiota disturbance and smoking as environmental risk factors for developing RA [36,51]. Therefore, a systemic break in tolerance occurs prior to onset of joint pathophysiology. Expansion of T cell-mediated autoimmunity through epitope spreading to additional self-antigens present in joints can then lead to onset of synovitis while formation of immune complexes between ACPAs and citrulline-containing antigens that further bind RF leads to abundant complement activation, thus potentiating the inflammatory response [33,35,36,42].

The primary manifestation of RA is autoimmune-mediated synovitis characterized by large-scale infiltration of leukocytes into the synovium, including autoreactive T cells (especially T helper (Th) cells Th1 and Th17) and B cells, macrophages, mast cells and neutrophils (the latter largely resident in the synovial fluid), accompanied by substantial release of inflammatory mediators, including cytokines, chemokines, eicosanoids, growth factors, vasoactive amines, matrix metalloproteinases (MMPs) and reactive oxygen species (ROS) [35,36,52]. Pro-inflammatory cytokines, particularly interleukin (IL)-6, induce the synthesis of acute-phase proteins (including C-reactive protein) involved in the acute-phase response. IL-6 as well as TNF-α, IL-17, IL-1 and transforming growth factor beta (TGF-β) can also induce osteoclastogenesis by enhancing the expression of receptor activator of nuclear factor kappa-B ligand (RANKL) in osteoblasts, FLSs, activated T cells and mature B cells. Binding of RANKL to its receptor, RANK, on monocytes and macrophages triggers differentiation to bone-resorbing osteoclasts, leading to bone erosion observed in RA [35,36,52].

In the inflamed RA synovium, activated FLSs adopt an apoptosis-resistant and aggressive proliferative phenotype leading to pannus formation with production of pro-inflammatory cytokines (e.g., TNF-α, IL-6 and IL-1β) and chemokines (e.g., IL-8, CCL2, CCL5 and CXCL10), extracellular matrix-degrading enzymes and pro-angiogenic factors resulting in chondrocyte apoptosis, cartilage matrix degradation and activation of endothelial cells [35,36,52]. Vascular endothelial growth factor (VEGF)-mediated angiogenesis and increase in vascular permeability promote further infiltration of leukocytes into the hypoxic synovium milieu, leading to synovial hyperplasia, joint swelling and systemic chronic pain [36]. Moreover, the invasive RA FLSs can migrate and infiltrate distant joints, resulting in symmetrical joint damage typical in RA [52].

Immune cells including CD4^+^ T, CD8^+^ T, NK and B cells are also involved in the complex pathogenesis of RA. Among them, CD4^+^ T cells stand out in relieving the pathological process of the disease. CD4^+^CD25^+^ regulatory T (T_reg_) cells, which have immunosuppressive functions, are part of the CD4^+^ T cell subset [53]. Expression of the specific nuclear transcription factor Foxp3 in CD4^+^CD25^+^ T_reg_ cells is a pivotal element for preserving inhibitory activity [53].

### 3.3. Treatment

Nowadays, RA can be effectively managed with different medication modalities. In addition, the adoption of a healthy lifestyle, including regular exercise, no smoking, reduced stress and an anti-inflammatory diet, such as the Mediterranean diet, rich in fruits, vegetables, whole grains, nuts, fish and olive oil, can also help in the treatment of disease [45]. Early diagnosis and treatment are essential to achieve remission or low disease activity. Initial treatment involves the use of disease-modifying anti-rheumatic drugs (DMARDs) able to delay or even halt disease progression, preventing radiographic progression and improving function and quality of life [26,33]. These are often used in combination with non-steroidal anti-inflammatory drugs (NSAIDs) or low-dose glucocorticoids (e.g., prednisone, prednisolone, dexamethasone, betamethasone and triamcinolone) to reduce pain and inflammation while the disease remains active. Glucocorticoids, although providing rapid symptomatic relief and useful in episodes of high disease activity (“flares”), are associated with serious long-term adverse events, including adrenal suppression [26,33]. DMARDs are immunosuppressive and immunomodulatory agents classified as either synthetic or biologic (Table 3). The former includes conventional synthetic DMARDs, like methotrexate (MTX), and targeted synthetic DMARDs, which are Janus kinase (JAK) inhibitors, for oral administration [26]. The Janus kinase inhibitors (JAKis) are orally available tsDMARDs that antagonize the activation of the intracellular cytoplasmatic enzymes JAKs, which control various biological functions, such as triggering the inflammatory cascade in immune cells. As a new type of DMARD, the JAKi targets a specific and critical pathway regarding the pattern of RA development and progress [54].

MTX is the most often used DMARD due to its efficacy to achieve remission or slow disease activity, and MTX plus a glucocorticoid is recommended as first-line RA therapy [26,33]. Insufficient response to this treatment within 3–6 months requires addition of a targeted synthetic DMARD or a biologic one [26]. Although JAKis have the maximum therapeutic effect when administered concomitantly with MTX, in patients where csDMARDs cannot be used as co-medication or in cases of poor prognostic condition, JAKis have shown a marked efficacy when used as monotherapy [55]. The recent trend is to start JAKis combined with MTX, followed by MTX reduction/discontinuation after achieving a sufficient therapeutic effect [55]. Following current therapeutic guidelines in the 2020 updated European League Against Rheumatism (EULAR) and the 2015 American College of Rheumatology guidelines, the combination of bDMARDs and tsDMARDs with conventional synthetic DMARDs (csDMARDs) is the most effective therapeutic approach for RA [55].

Biologic DMARDs are highly specialized genetically engineered proteins for parenteral administration that target specific soluble inflammatory mediators, immune cells or signaling pathways involved in RA pathogenesis [26,33,47]. These biological response modifiers include TNF inhibitors, IL-6 receptor (IL-6R) inhibitors, T cell co-stimulation inhibitors (abatacept, binds to CD80/CD86 on antigen-presenting cells, modulating T cell activation) and B cell-depleting agents (rituximab, anti-CD20 monoclonal antibody), being an effective second-line treatment for pathogenesis [26,33,47]. IL-1 inhibitors, such as the IL-1 receptor antagonist (IL-1Ra) anakinra, have also been licensed for RA treatment. However, lower efficacy compared with other biologic DMARDs and a dose schedule requiring daily subcutaneous injections do not recommend its use [26,33,47].

DMARDs are associated with several adverse events, including malignancies, major adverse cardiovascular events, venous thromboembolism and increased risk of serious infections (more frequent with biologics), including tuberculosis reactivation [26,33]. Safety aspects, patient clinical history and cost of therapy must be considered in DMARD selection, though the introduction of biosimilar DMARDs contributed to a reduction in the price of biologics [26]. DMARDs may be tapered (by reducing the dose or increasing the interval between doses) during sustained remission but should not be stopped [26,33].

## 4. Animal Models of Rheumatoid Arthritis

Animal models of RA are valuable resources for studying the disease pathogenesis and testing novel anti-RA drug candidates. Both spontaneous and induced experimental models have been used in RA research. Spontaneous RA can be modeled using genetically modified mice, such as human TNF transgenic mice, IL-1Ra knockout mice, double transgenic K/BxN (showing cross-reactive autoantibodies against glucose-6-phosphate isomerase) and SKG transgenic mice [56,57]. The latter develop T cell-mediated chronic and progressive autoimmune polyarthritis, spontaneously and upon stimulation with intraperitoneal zymosan injection [56,57].

Antibodies, antigens and adjuvants are usually used to induce RA in animal models [57]. The first established animal model of RA was adjuvant arthritis (AA) induced in rats by a single subcutaneous injection of complete Freund’s adjuvant (CFA), consisting of a suspension of heat-killed *Mycobacterium tuberculosis* in mineral oil injected into the rat’s hindfoot or tail root [56,57]. CFA induces polyarthritis 10–45 days after immunization due to T cell response to the mycobacterial heat shock protein Hsp65. Additionally, some adjuvants without immunogenic properties can also induce arthritis in susceptible animal strains, including incomplete Freund’s adjuvant (IFA), which lacks mycobacteria [56,57].

In the antigen-induced arthritis (AIA) model, an antigen, such as ovalbumin or bovine serum albumin, is intra-articularly injected into the knee joint of animals (mice, rats or rabbits) after previous sensitization by subcutaneous injection of the protein emulsified in CFA [56,57]. Boosting of the immune response is achieved by concomitant intraperitoneal administration of heat-inactivated *Bordetella pertussis*. AIA is a T cell-dependent monoarthritis model and T cell-mediated flares can be induced by local or systemic rechallenge with low-dose antigen [57]. Modified antigens, e.g., methylated proteins, are used to induce chronic arthritis [56,57].

Collagen-induced arthritis (CIA) is the gold standard *in vivo* model of RA, mainly characterized by breach of tolerance and production of autoantibodies against self-collagen, resembling human RA [56,57,58]. Typically, susceptible mice strains are immunized with bovine, murine or chicken CII emulsified in CFA and injected intradermally into the mouse’s tail [58]. Rats are generally susceptible to adjuvant-induced arthritis, after being immunized with an emulsion of CII in IFA subcutaneously injected at the base of the tail [58]. The development of CIA is associated with both B cell and T cell responses with production of anti-CII antibodies and collagen-specific T cells [56]. A booster immunization with an emulsion of CII in IFA is frequently applied following primary immunization (on the 14th or 21st day for mice and the 7th day for rats) to ensure high CIA incidence [58]. Clinical signs of polyarthritis appear 21–28 days (mice) or 2–3 weeks (rats) after the first immunization, depending on the strain [58]. This model has also been expanded to non-human primates [57].

On the other hand, in the collagen antibody-induced arthritis (CAIA) model, a simple mouse model of RA, arthritis is induced by tail vein administration of a cocktail of anti-CII monoclonal antibodies, usually followed by intraperitoneal injection of lipopolysaccharide (LPS) to enhance the incidence and severity of the disease [57]. The CAIA model has several advantages over the classic CIA model, such as rapid disease onset (24–48 h after LPS injection), synchronicity and the capacity to use genetically modified mice, including gene knockout and transgenic mice [57].

Other experimentally induced inflammatory models of RA include streptococcal cell wall-, proteoglycan- and zymosan-induced arthritis. A single intraperitoneal injection of streptococcal cell wall peptidoglycan–polysaccharide polymers induces a cycle of exacerbation and remission of inflammatory arthritis in the peripheral joints of rodents [56,57]. Mice immunized with intraperitoneal injection of human proteoglycans isolated from cartilage of RA patients submitted to joint replacement surgery develop autoantibodies and inflammatory polyarthritis [57]. Intra-articular injection of zymosan, a polysaccharide from the cell wall of *Saccharomyces cerevisiae*, induces chronic proliferative inflammatory monoarthritis following complement activation via the alternative pathway [56,57]. Although none of the developed experimental models can perfectly reproduce the pathophysiology of human RA, they are useful tools for identification of new targets and development of novel therapies, as exemplified by cytokine inhibitors [56].

## 5. Triterpenes and Some Biosynthetic Considerations

Triterpenes are a large and structurally diverse group of natural compounds, widely distributed through the plant kingdom [59]. They can be classified as primary metabolites, e.g., phytosterols that are structural constituents of the cell membranes and ubiquitous in all plant organisms, and secondary metabolites that are generally restricted to some plant families and genera [21,60]. According to the isoprene biogenetic rule, triterpenes derive from an all-*trans* squalene C30 precursor [21]. Squalene is derived from two farnesyl diphosphate units (C15) by a tail-to-tail coupling catalyzed by squalene synthase. Cyclization of squalene proceeds in the vast majority of cases, by its oxidation to squalene 2,3-epoxide catalyzed by squalene epoxidase. The polycyclic structure adopted from squalene depends on the conformation in which the squalene chain can be folded on the oxidosqualene cyclase enzyme surface, into chair or boat conformations, or with a part remaining unfolded. The formation of the polycyclic triterpenic scaffold can be rationalized by a sequence of cyclizations, usually initiated by acid-catalyzed ring opening of the squalene epoxide and through a series of carbocation intermediates in a stepwise sequence, giving rise to more than 200 distinct triterpene skeletons [21,61,62]. A deeper explanation of triterpene biosynthesis is beyond the scope of this work and further details, including genes and enzymes regulating the biosynthetic pathways, can be found in several excellent reviews [20,59,60,61,62].

**Figure 1:**
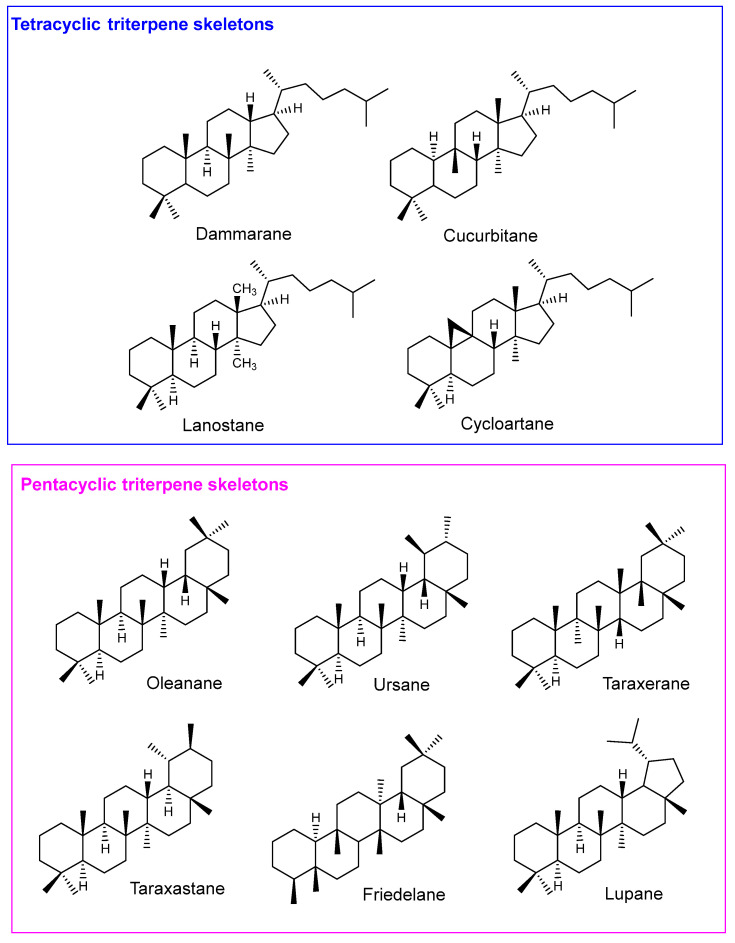
Structures of the main tetracyclic and pentacyclic triterpene skeletons.

Most triterpenes have tetracyclic (C6-C6-C6-C5; e.g., dammarane, cucurbitane, lanostane and cycloartane types), and pentacyclic (C6-C6-C6-C6-C5 or C6-C6-C6-C6-C6; e.g., oleanane, ursane, lupane, friedelane, hopane and taraxastane types) scaffolds (Figure 1), but acyclic, monocyclic, bicyclic and hexacyclic structures have also been isolated [21]. Triterpenes may have a variety of oxygenated functional groups and unsaturations, giving rise to a high number of structurally diverse compounds. They can also be found in either free or glycosidic form (saponins), where one or more sugar residues are covalently linked to the triterpenic nucleus. Saponins are amphiphilic compounds due to the lipophilic sapogenin and the hydrophilic sugar side chain(s), forming stable soap-like foams in solution [21]. Even though saponins are highly toxic when injected in the bloodstream, causing hemolysis of the red cells by increasing the permeability of the plasma membrane, they are relatively harmless when taken orally. The toxicity is minimized after ingestion by low absorption and by the acid-catalyzed hydrolysis that releases the aglycone and the molecules of sugar [21].

## 6. Triterpenes with Rheumatoid Arthritis-Related Effects

**Figure 2:**
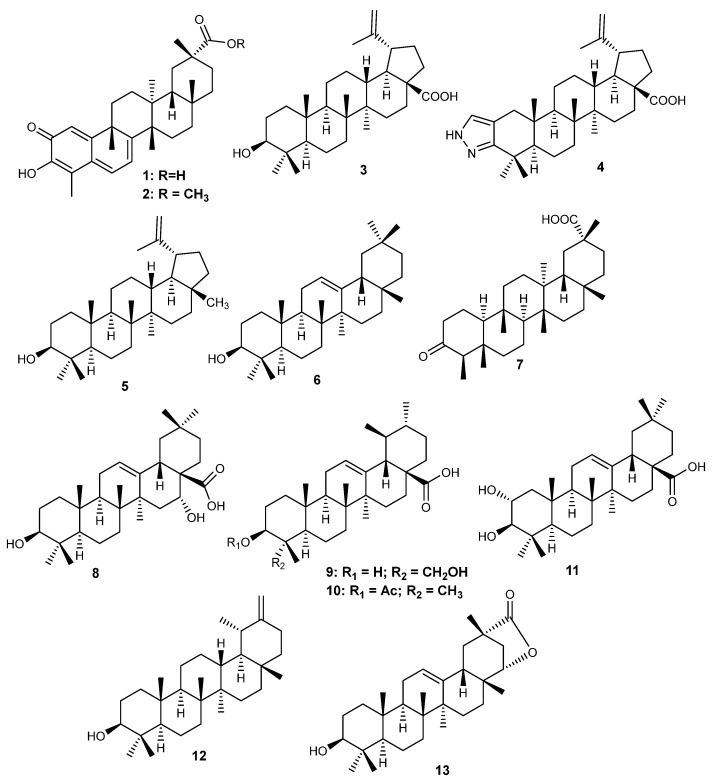
Structures of pentacyclic triterpenes (**1**–**13**) with activity on RA.

**Figure 3:**
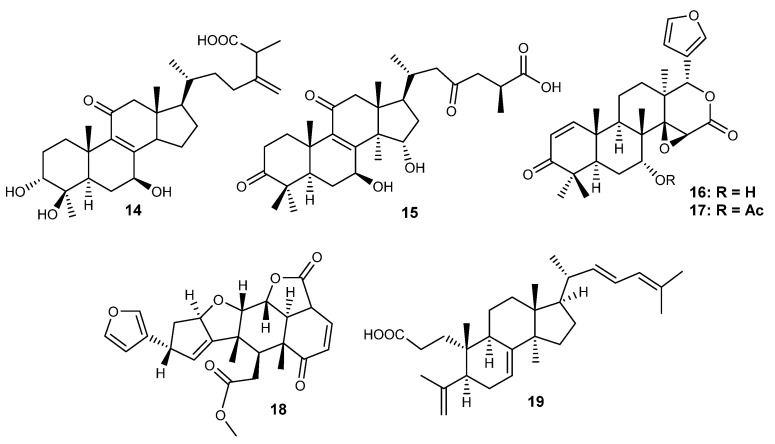
Structures of tetracyclic (**14**–**15**) and rearranged triterpenes (**16**–**19**) with activity on RA.

**Figure 4:**
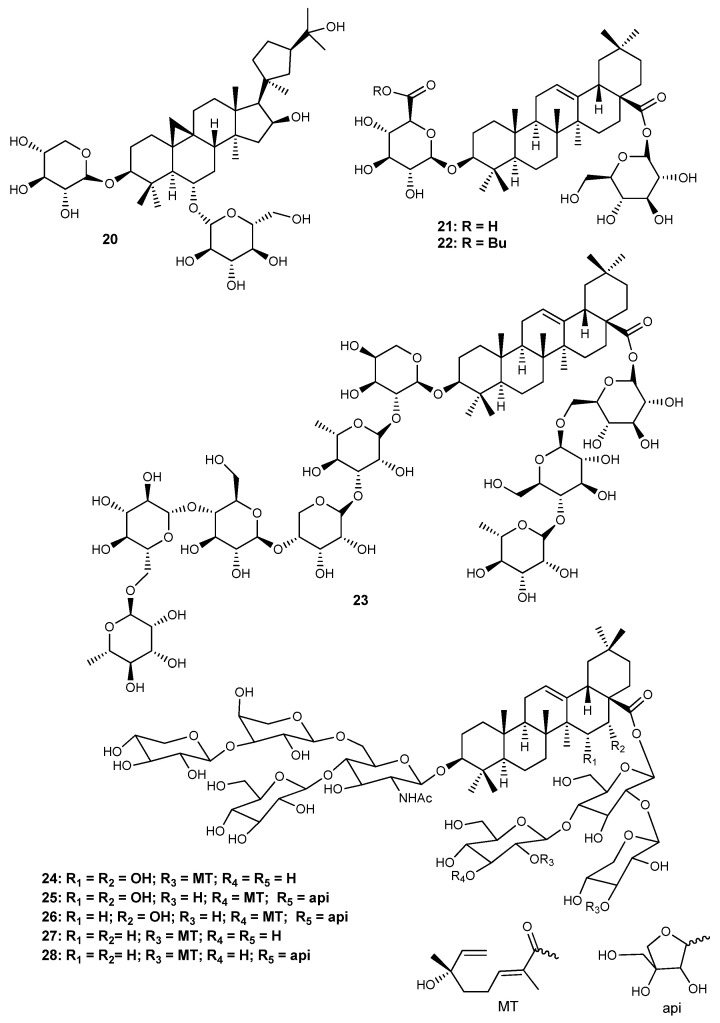

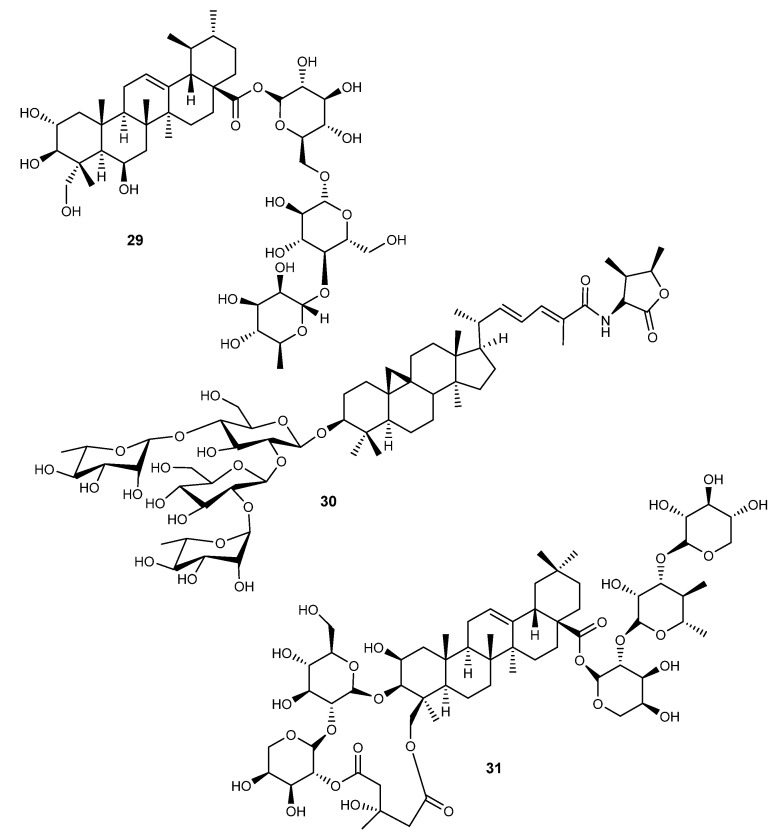
Structures of triterpenic saponins (**20**–**31**) with activity on RA.

**Figure 5:**
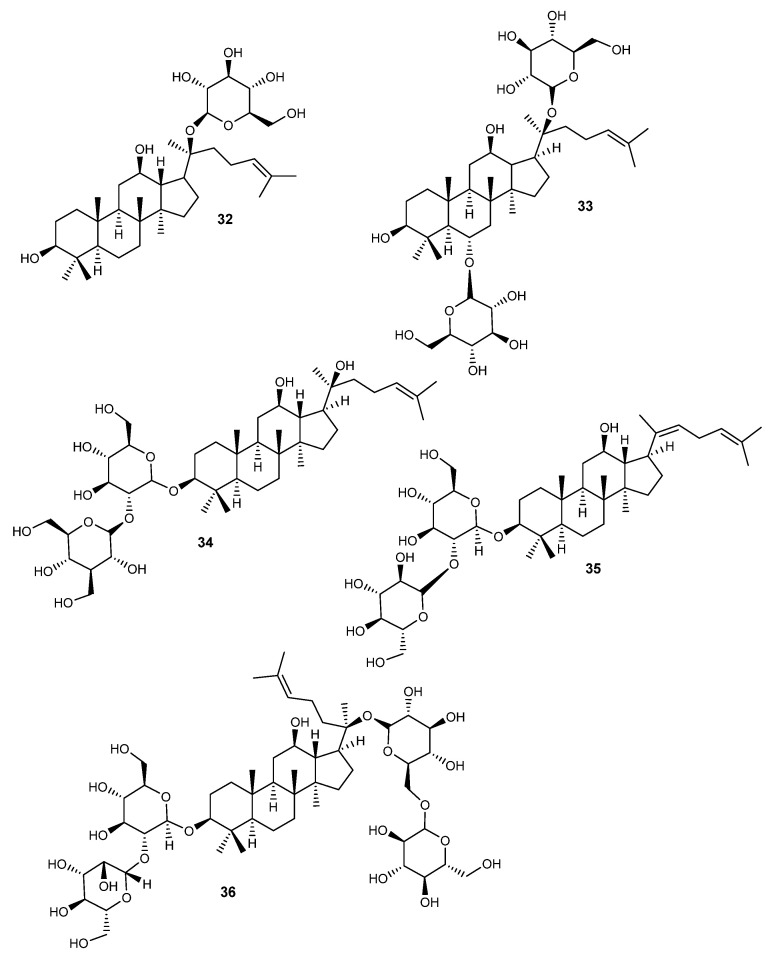
Structures of ginsenosides (**32**–**36**) with activity on RA.

Herein, 36 triterpenic compounds with RA-related *in vitro* and/or *in vivo* effects reported in the literature from 2017 to 2022 are presented (Figure 2, Figure 3, Figure 4 and Figure 5 and Table 4, Table 5 and Table 6). The triterpenes are divided into three major classes: pentacyclic triterpenes (Figure 2 and Table 4), tetracyclic and rearranged triterpenes (Figure 3 and Table 5) and triterpenic saponins (Figure 4 and Figure 5 and Table 6).

### 6.1. Pentacyclic Triterpenes

Celastrol (**1**), also known as tripterine, is a nor-triterpene quinone methide with the friedelane skeleton found in *Tripterygium wilfordii*, known as “Thunder God Vine”, a vine commonly grown is southeast China and used in traditional Chinese medicine for the treatment of RA and other autoimmune and inflammatory diseases [63]. Recent studies suggest that NLRP3 inflammasome-induced inflammation is involved in the pathogenesis of RA [47]. Celastrol (**1**) treatment significantly reduced the secretion of IL-1β and IL-18 in the serum of CFA-induced rats and in supernatants of human mononuclear macrophages (THP-1 cells) due to inhibition of the NF-κB pathway and hindering of NLRP3 inflammasome activation [63]. **1** also suppressed ROS production induced by LPS and adenosine triphosphate (ATP) in THP-1 cells [63] and prevented NLRP3 inflammasome activation *in vitro* by inhibiting complex formation between NLRP3 and ASC adaptor protein [64], essential for recruitment of caspase-1 and maturation of IL-1β. **1** also inhibited TNF-α-induced proliferation of FLSs, enhanced autophagosome levels and expression of autophagy-related proteins (LC3, p62 and Beclin-1) and increased the LC3-II/LC3-I ratio [65]. Furthermore, the autophagy inhibitor 3-methyladenine significantly reversed effects of **1** on the expression of autophagy-related proteins [65]. In CIA mice, **1** attenuated disease severity via upregulation of autophagy through inhibition of the PI3K/Akt/mTOR axis [65]. Autophagy dysregulation has been implicated in several autoimmune diseases, including RA. Enhanced autophagy contributes to RA FLS hyperplasia and apoptosis resistance, production of citrullinated peptides, osteoclastogenesis and bone resorption, resulting in severe bone and cartilage damage [66].

The co-administration of **1** and diclofenac has been routinely used in Chinese medicine for the treatment of RA. In order to shed light on the possible interaction potential of the two drugs, Wang et al. studied the *in vivo* effects of diclofenac on the pharmacokinetic profiles of **1** in rats [67]. When co-administered, several pharmacokinetic parameters significantly change, in particular, the *C*_max_ and the AUC_0_ of **1** decreased from 66.93 ± 10.28 to 41.25 ± 8.06 μg/L and 765.84 ± 163.61 to 451.33 ± 110.88 (μg × h/L), respectively. On the other hand, *T*_max_ increased from 6.05 ± 1.12 to 7.82 ± 1.15 h, and oral clearance increased from 1.29 ± 0.15 to 2.27 ± 0.31 L/h/kg. Moreover, it was found that the efflux ratio of **1** across the Caco-2 cell model increased when co-administered with diclofenac. In this way, the authors concluded that diclofenac could decrease the exposure of **1** in rats. It was also suggested that this effect could be carried out by decreasing the intestinal absorption of celastrol (**1**) through induction of P-glycoprotein (P-gp) activity [67].

To evaluate the progression of the disease and the response of RA patients to treatment, several biomarkers have been used, such as RF and ACPAs, although they can also be found in other autoimmune diseases. In this way, Dudics et al. studied the micro-RNA profile of immune (lymphoid) cells of arthritic Lewis rats and celastrol (**1**)-treated arthritic rats, in order to evaluate its ability as a novel RA biomarker [68]. Using combined miRNA–microarray technology and bioinformatics-based analysis, it was found that eight specific miRNAs (miR-22, miR-27a, miR-96, miR-142, miR-223, miR-296, miR-298 and miR-451) and their target genes are crucially involved in functional pathways for RA pathogenesis. In particular, miR-22, miR-27a, miR-96, miR-142, miR-223, miR-296, miR-298 and miR-451 were modulated by celastrol (**1**) treatment. Through the quantitation of these miRNAs in serum samples of control, arthritic and celastrol (**1**)-treated rats, in the peak phase of adjuvant-induced arthritis, it was found that miR-142, miR-155, miR-212 and miR-223 levels were higher in arthritic *vs*. control rats, further validating their value as circulating biomarkers to assess arthritis progression and response to therapy [68].

Fang et al. aimed at studying the effect of **1** on activated RA FLSs obtained from synovial biopsies of human RA patients [69]. Several assays were carried out in order to assess proliferation, invasion and expression of pro-inflammatory cytokines and to screen for differentially expressed genes. The authors found that **1** significantly modulated the RA–FLS activation status by reducing the proliferation and invasion of the cells. Moreover, a change in the expression of several chemokine genes, including CCL2, CXCL10, CXCL12, CCR2 and CXCR4, was also observed. This finding could be useful for therapy since chemokines could be responsible for the arthritis pain by promoting leukocyte infiltration and synoviocyte proliferation and activation. In particular, the release of CCL2 and CXCL12 proteins from RA FLS cells was significantly downregulated by celastrol (**1**) treatment. Celastrol (**1**) treatment also diminished the activation and translocation of NF-κB p65, which is known to participate in the regulation of many cytokines, adhesion molecules, chemokines, receptors and adaptive enzymes in arthritis [69].

Inhibition of oxidative stress underlies the improvement observed in CIA rats treated with **1** (1 mg/kg) in a study carried out by Gao et al. [70]. **1** enhanced the superoxide dismutase activity and significantly inhibited the levels of malondialdehyde, superoxide anions and NADPH oxidase activity [70]. Reduction of arthritis scores and spleen and thymus indexes was also observed, as well as the suppression of serum levels of TNF-α, IL-1β, IL-6 and interferon gamma (IFN-γ), which could be attributed to the downregulation of inflammatory mediators [70].

The mechanistic complexity of **1**, due to its multiple targets, was analyzed by Song et al. by employing a network pharmacological approach. The authors identified probable molecular targets of the compound and the interaction pathways related to their roles, investigating the networks formed by those pathways [71]. Using a web-based bioinformatics application (ingenuity pathway analysis), pathways and networks were built grounded in the functions of the human genes appertaining to RA and the selected potential targets. The networks comprised cell movement, immune cell trafficking, hematological system development and function, inflammatory response, connective tissue disorders, organismal injury and abnormalities and cell-to-cell signaling and interactions. Results indicated that MMP-9, COX-2, c-Myc, TGF-β, c-JUN, JAK-1, JAK-3, IKK-β, SYK, MMP-3, JNK and MEK1 were the direct targets of **1** in RA. Being high-degree nodes in RA-associated networks probably affected by **1,** COX-2, IKK-β, JNK and MEK1 were selected for docking studies [71]. Results of the pathway analysis obtained by Song et al. suggested that **1** can regulate the functions of Th1 and Th2 cells, fibroblasts, macrophages and endothelial cells, which would explain its therapeutic effects against RA [71].

Pristimerin (**2**) is the celastrol methyl ester, a natural triterpene found in plants of the Celastraceae and Hippocrateaceae families. In TNF-α-stimulated human RA FLSs, treatment with **2** decreases cell viability and migration in a dose-dependent manner [72]. According to cell metabolomics analysis, the effects involved phospholipid and fatty acid biosynthesis, glutathione metabolism and amino acid metabolic pathways [72]. *In vivo*, compound **2** ameliorated arthritis symptoms and reduced serum levels of TNF-α and NO and synovial expressions of p-Akt and p-ERK in the CFA-induced arthritis rat model. Network pharmacology analysis showed that the effects were mediated through the MAPK/ERK1/2 and PI3K/Akt pathways and direct binding to TNF-α [72].

The effects of betulinic acid (**3**) on the proliferation, migration and inflammatory response of RA FLSs were studied by Wang and Zhao [73]. Compound **3** inhibited the proliferation, migration and invasion of RA FLSs in a dose- and time-dependent manner at non-cytotoxic concentrations (5–20 μM). It also decreased MMP expression and inhibited the production of TNF-α-induced inflammatory cytokines, namely of IL-6 and IL-8. The PI3K/Akt signaling pathway plays a significant role in regulating inflammation, proliferation and migration of RA FLSs and in signal activation of NF-κB, being highly expressed in the synovial tissues of RA patients. Betulinic acid (**3**) also avoided activation of the Akt/NF-κB pathway and can be considered a potential therapeutic agent for the treatment of RA [73].

Huimin et al. explored the protective effects of **3** on CFA-induced rats, observing the significant inhibition activity of the drug regarding the arthritis index, toe swelling, joint pathology and hemorheology [74]. Serum and synovial levels of Il-6, IL-1β and TNF-α were improved following treatment with **3**. Since Rho and Rho-associated protein kinase (ROCK) control the production of inflammatory cytokines, the anti-inflammatory mechanism of **3** was investigated through the Rho/ROCK/NF-κB activation by treating rats with fasudil (a ROCK inhibitor). Protein levels of RhoA, ROCK1 and ROCK2 were downregulated, leading to the blockage of phosphorylation of IKKα, IKKβ, lκB and NF-κB. The results provided information about the mechanism of compound **3** on RA, which may be related to the downregulation of ROCK/NF-κB signaling pathways [74].

Since RA FLSs display an aggressive phenotype, which is linked to cartilage and bone destruction, Li et al. examined the effects of **3** on the migration and invasion of RA FLSs (prepared from synovial tissue specimens of diagnosed RA patients), seeking a mechanistic understanding of the therapeutic potential of **3** [75]. Treatment with **3** restrained the migratory and invasion capacity of RA FLSs and decreased the formation of actin stress fibers and actin cytoskeleton score [75]. Considering the TNF-α-induced RA FLSs, treatment with **3** led to a significant decrease in the mRNA expression of IL-1β, IL-6, IL-8 and IL-17A, as well as to a decrease in phosphorylated IKK, IκBα and NF-κB and to a reduction of the NF-κB accumulation. These results suggest that the inhibition of NF-κB signaling pathways by **3** causes the inhibition of migration, invasion, actin cytoskeleton reorganization and interleukin expression of RA FLSs [75].

RA is highly associated with increased risk of cardiovascular disease, with RA patients being almost twice as likely to develop heart disease as compared with the general population [76]. Besides the traditional risk factors, chronic inflammation associated with RA appears to promote atherosclerosis, and in both diseases similar pathophysiologic processes are recognized, including increased expression of cellular adhesion molecules, pronounced infiltration by macrophages and Th1 cells, neovascularization and collagen degradation mediated by MMPs [77]. Statins are HMG CoA reductase inhibitors widely used for treatment of hyperlipidemia and prevention of cardiovascular disease, and their anti-inflammatory properties have been proved to be associated with several molecular mechanisms such as suppression of chemokine and pro-inflammatory cytokine synthesis, MMP inhibition, reduced MHC-II expression induced by IFN-γ and reduced expression of CD40 on macrophages and other smooth muscle cells [78,79]. The synergist effect of oral co-administration of **3** (2 mg/kg) and fluvastatin (5 mg/kg) was studied *in vivo* using a CIA rat model, and several physical, morphological and biochemical parameters were collected [80]. Combined treatment with **3** and fluvastatin showed a decrease in the severity of arthritic index values and inhibition of paw edema (89%) after 60 days when compared with the single administration of the drugs (80% and 74%, respectively) or the control group without treatment. A reduction of RF, C-reactive protein, total lipids and ACPAs, as well as an increased activity of catalase, superoxide dismutase and glutathione peroxidase enzymes, in the different tissues was also observed in the rats treated with a combination of both drugs. Moreover, it was also found that the expression of the anti-inflammatory cytokine IL-10 was increased in the co-treated group, while the expression of Toll-like receptor (TLR) 2 and TLR4, IL-1β, TNF-α, IFN-γ, cell adhesion molecules and nuclear translocation of NF-κB in the aorta decreased, when compared to the single-treated groups [80].

Taking into consideration that betulinic acid (**3**) can be regarded as a lead compound for further development of potential anti-inflammatory agents, several derivatives having heterocyclic rings fused at C-2 and C-3 were synthesized and assayed as inhibitors of osteoclast differentiation and bone resorption [81]. The most potent compound, pyrazole derivative **4**, exhibited potent inhibitory activity on RANKL-induced osteoclast formation (IC_50_ = 0.09 μM), being 200-fold more active than the parent triterpene **3**. In a later work, Chen et al. studied the modulation activity of **4** on T cell differentiation and proliferation and potential anti-rheumatic effects in a CIA mouse model [82]. When compared to the control group that received no treatment, the severity of symptoms was significantly attenuated in treated mice, that showed a mean arthritis score of 2.63 on day 41 (control group: 6.88). Further radiological and histopathological analysis corroborated these findings, since considerably less articular damage was observed and arthritis cartilage destruction and inflammatory cell infiltration were highly decreased, possibly due to inhibition of Th1 and Th17 differentiation, enhanced IL-4, IL-10, IL-13 expression and increased CD4^+^ Foxp3^+^ cells [82].

Lupeol (**5**), a lupane triterpenoid with antioxidant and anti-inflammatory properties found in many edible fruits and vegetables, inhibited PI3K/Akt signaling in CIA rats [83]. Lupeol significantly reduced paw edema, reverted the high levels of biochemical markers (RF, C-reactive protein and ceruloplasmin) and pro-inflammatory mediators (TNF-α, IL-6 and PGE2) in the rat serum and enhanced apoptosis by downregulating Bcl-2 protein expression while upregulating Bax, caspase-3 and caspase-9 [83]. However, the overall effects were inferior to those of indomethacin, the NSAID used as positive control [83].

β-amyrin (**6**) and polpunonic acid (**7**) are found in the root bark of *Ziziphus abyssinica* (Hochst Ex A. Rich), a recognized medicinal plant widely distributed in the tropical regions of the world, showing antioxidant, anti-bacterial and anti-plasmodial activities, among others [84]. Henneh et al. were able to isolate them as pure chemical entities and determine their absolute configuration, examining possible therapeutic effects in RA in a CFA-induced arthritis rat model [84]. Compounds **6** and **7** (at equal doses) reversed the changes induced in the RA model (considering body weight, paw thickness, erythema and arthritic index). Histopathological examinations of rat hind paws showed a significant reduction of cartilage erosion and subchondral cyst and Weichselbaum’s lacunae formation, with an effect dependent on the type of compound and the doses of administration. There was also evidence of bone remodeling and decreased bone cavitation after treatment with both compounds, most pronounced for **6** [84].

Echinocystic acid (**8**) isolated from the bark of *Albizia julibrissin* Durazz was able to ameliorate arthritic symptoms induced in transgenic SKG mice after a single intraperitoneal injection of zymosan. The treatment with **8** reduced inflammatory cell infiltration, pro-inflammatory cytokine levels, synovial hyperplasia and bone loss in mouse paw tissues [85]. These effects have been attributed to inhibition of both IL-6- and TGF-β-induced Th17 cell differentiation, namely by suppression of phosphorylation of STAT3. In TNF-α-activated human RA FLSs (MH7A cells), administration of **8** reduced both protein and mRNA expression of inflammatory cytokines (IL-6 and IL-1β) by downregulating MAPK and NF-κB signaling pathways [85].

Bone homeostasis depends on the balance between osteoclast-mediated bone resorption and osteoblast-mediated bone formation. Excessive osteoclast activity has been associated with RA, osteoarthritis, osteoporosis and other bone-related diseases [36,52,86]. 23-Hydroxyursolic acid (**9**) isolated from *Viburnum lutescens* was found to inhibit RANKL-induced osteoclastogenesis *in vitro* by decreasing the number of tartrate-resistant acid phosphatase (TRAP)-positive osteoclasts and F-actin ring formation [87]. Actin ring formation is a characteristic marker of bone resorption activity of mature osteoclasts. Compound **9** also inhibited RANKL-induced phosphorylation of ERK and JNK, IκBα degradation, c-Fos expression, activation of the nuclear factor NFATc1 and expression of its target genes [87]. Oral administration of **9** to mice conferred protection against LPS-induced osteoclast formation and bone loss [87].

The study conducted by Lee et al. compared the *in vitro* and *in vivo* effects of ursolic acid-3-acetate (**10**) and dexamethasone, using TNF-α-stimulated human FLSs and a murine model of RA [88]. The treated rats showed a decrease in clinical symptoms, including clinical arthritis score, disease incidence and paw thickness, which were confirmed by microPET imaging. A decrease in serum IgG1 and IgG2a levels was also observed. Characteristic RA histological and radiological changes, such as hyperplasia, pannus formation, cartilage destruction and bone erosion in the joint, were improved, with results comparable to the anti-inflammatory drug dexamethasone. On the other hand, the *in vitro* studies revealed a reduction of Th1/Th17 phenotype CD4^+^ T lymphocyte expansion, pro-inflammatory cytokines (IL-1β, IL-6, IFN-γ and IL-17) and MMP-1/3 production in the knee joint tissue and RA synovial fibroblasts, through the downregulation of IKKα/β, ΙκBα and NF-κB [88].

Maslinic acid (**11**), a pentacyclic triterpenoid found in olive (*Olea europaea*) fruit, displays a vast number of therapeutic properties, including preventing and mitigating arthritis in animals and humans, particularly in relation to knee joint arthritis symptoms [89]. Using the CAIA mouse model of RA, Shimazu et al. clarified the molecular mechanisms implicated in the anti-arthritic properties of **11**. Arthritis symptoms were mitigated, and the gene expression of inflammatory cytokines in synovial membranes was inhibited downstream of NF-κB signaling, with **11** also inactivating the TLR signaling pathway. Treatment of CAIA mice with **11** (200 mg/kg) downregulated the expression of the mRNA encoding LTA4 hydrolase, which catalyzes the hydrolysis of LTA4 to LTB4, a chemotactic factor whose overproduction is involved in RA. **11** suppressed the production of LTB4 by acting through the glucocorticoid receptor, as expression levels of several genes controlled by this receptor were altered by **11** [89]. Upregulation of the mRNAs encoding MMP-2 and MMP-9 was observed, along with the upregulation of the expression levels of transcripts encoding tissue inhibitor of metalloproteinases (TIMP)-1, TIMP-2 and TIMP-4, where the proteinase/inhibitor imbalance can facilitate proteolysis in the cartilage of arthritis [89]. The anti-arthritis efficacy of compound **11** thus appears to be grounded in the suppression of synovial inflammation through the inactivation of TLRs, the downregulation of leukotrienes via the glucocorticoid receptor and the promotion of tissue formation with the repair of damaged cartilage [89].

Taraxasterol (**12**) is a taraxastane-type triterpenoid mostly isolated from Chinese medicinal *Taraxacum officinale*, exhibiting anti-inflammatory and antioxidant activities in several disorders [90]. Literature reports have been pointing to its ability to lower pro-inflammatory cytokines and mediators in LPS-induced RAW 264.7 cells *in vitro* and in the ovalbumin-induced asthma mouse model [90]. *In vitro* and *in vivo* studies of **12** in IL-1β-stimulated human RA FLSs and CIA mice, respectively, allowed Chen et al. to investigate the anti-inflammatory effects and subjacent mechanisms of **12** on RA [90]. Since the inflammatory responses in RA FLSs are mostly modulated by NF-κB and the NLRP3 inflammasome [90], the inhibition of NF-κB/NLRP3 pathways is therefore a potential therapeutic approach in RA management. In fact, **12** suppressed NF-κB activation in human RA FLSs, inhibiting the IL-1β-induced IκB degradation and nuclear translocation of p65 in the studied cell line. Results showed that **12** can modulate TGF-β-activated kinase 1 (TAK1) activation (which in turn regulates NF-κB activation), probably exerting its anti-inflammatory activity by modulating the TAK1/IκB/IKK pathway in human RA FLSs [90]. Compound **12** suppressed the expression of NLRP3 inflammasome (reported to be well associated with NF-κB signal transduction) and its modulators, such as TXNIP and ACS, both in human RA FLSs and CIA mice, thereby decreasing cleaved caspase-1 levels; thus, anti-inflammatory effects of **12** could be related to the inhibition of NLRP3 inflammasome signaling. Treatment of CIA mice with **12** mitigated joint destruction and other clinical RA manifestations, downregulated NF-κB and reduced the IL-1β-induced expressions of TNF-α, IL-6, IL-8, MMP-1 and MMP-3 [90].

Macrophage plasticity produces different functional phenotypes in reaction to specific stimuli. Macrophages can be polarized into the classical M1 or the alternative M2 phenotypes. Classically activated (M1) macrophages, induced by LPS or Th1 cytokines IFN-γ and granulocyte–macrophage colony-stimulating factor (GM-CSF), express MHC-II, inducible nitric oxide synthase (iNOS) and co-stimulation molecules like CD80 and CD86 for effective T cell antigen presentation and secrete pro-inflammatory cytokines (e.g., TNF-α, IL-1β, IL-6, IL-12 and IL-23) as well as NO and ROS which are essential for killing intracellular pathogens [36,91]. Alternatively, activated (M2) macrophages, stimulated mainly by Th2 cytokines IL-4 and IL-13 and by macrophage colony-stimulating factor (M-CSF), express mannose receptor CD206, IL-4 receptor, arginase 1 and peroxisome proliferator-activated receptor gamma (PPARγ) and produce anti-inflammatory cytokines (e.g., IL-10 and TGF-β) and trophic polyamines involved in tissue repair [36,91]. The M1/M2 polarization is imbalanced in RA, with higher expression of M1 macrophages in the synovial fluid of RA patients, which promotes osteoclastogenesis [86,91]. ACPAs in the RA synovial fluid can induce interferon regulatory factor 5 (IRF5), leading to increased polarization of peripheral blood monocytes into the M1-like phenotype and thus increasing the M1/M2 ratio [91]. Glucocorticoids and some DMARDs like MTX act by repolarizing M1-like macrophages of RA patients into the M2-like state [86,91]. Wilforlide A (**13**), a pentacyclic triterpenoid from *Tripterygium wilfordii* Hook F, delays the development of RA in CIA mice, inhibiting iNOS production (an M1 surface marker), pro-inflammatory M1 cytokines and chemokines in the mouse synovium [92]. Similarly, *in vitro* results showed that **13** hindered macrophage chemotaxis and M1 polarization in LPS/IFN-γ-stimulated THP-1 cells presumably through inactivation of the TLR4/NF-κB signaling pathway [92].

**Table 4:** Pentacyclic triterpenes with *in vitro*/*in vivo* RA-related effects.

Pentacyclic Triterpene	Cell Model/Animal Model/Dosage	Effects and Mode of Action	Ref.
Celastrol (**1**)	TNF-α-stimulated FLSs; pre-treated with **1** (0, 25, 50 or 100 nM) for 2 h and stimulated with TNF-α (10 ng/mL) for 48 h CIA in male DBA/1 SPF grade mice; intragastric administration of **1** (0, 0.5, 1 or 2 mg/kg/day), vehicle (0.5% CMC-Na) or MTX (2 mg/kg/day), on days 28 to 56 post-immunization	*In vitro* inhibition of TNF-α-induced proliferation of FLSs Decrease in p-mTOR, PI3K and p-AKT levels Increase in autophagosome levels, LC3-II/LC-I ratio and Beclin-1 expression, *in vitro* and *in vivo* *In vivo* inhibition of the production of pro-inflammatory cytokines TNF-α and IL-1β Reduction of protein levels of PI3K, p-AKT, p-mTOR and p62 in joint tissue, thus ameliorating paw swelling and hind paw bone damage in CIA mice	[65]
	LPS/ATP-stimulated human macrophages (THP-1 cells); incubation with PMA (100 nM) for 48 h and treated with **1** (0, 12.5, 25 or 50 nM) or dexamethasone (50 nM) for 1 h prior to incubation with LPS (1 µg/mL) for 24 h followed by ATP (5 mM) stimulation for 30 min AA in male SD rats; injected with CFA in the left hind joint on day 1 and treated with **1** (0.5 or 1 mg/kg) or vehicle (0.9% saline), i.p., daily, from day 9 up to day 30	Reduction of joint swelling, arthritis index score, inflammatory cell infiltration and synovial hyperplasia in CFA-induced rats Decrease in levels of IL-1β and IL-18 in the rat serum and supernatants of THP-1 cells exposed to **1** Inhibition of ROS production, blocking of NF-κB signaling and hindering the activation of the NLRP3 inflammasome	[63]
	CIA male Wistar rats; intradermal injection twice at the base of tail with BTIIC emulsion with CFA (1 mg/mL); day 0 (200 μL) and day 7 (100 μL). Experiment I: CIA rats treated i.p. with **1** (1 mg/kg/day) or vehicle. Experiment II: CIA rats treated i.p. with **1** (1 mg/kg/day) and Ad-Nox4 (1 × 1010 TU/mL; tail vein) for 28 days	Significant reduction of paw edema and arthritis scores. Improvement of the spleen and thymus indexes Reduction of TNF-α, IL-1β, IL-6, IFN-γ levels in CIA rats Increase in superoxide dismutase activity; reduction of malondialdehyde and superoxide anions levels and NADPH oxidase activity Potential therapeutic effects on RA may be ascribed to downregulation of inflammatory cytokine levels and attenuation of oxidative stress	[70]
	Caco-2 cell line; treated with increasing concentrations of **1** (1–10 μM for viability assays; 2 μM for P-gp efflux) Male Sprague Dawley rats administered with **1** (1 mg/kg, control group) or both **1** (1 mg/kg) and diclofenac (10 mg/kg)	Significant change in several pharmacokinetic parameters suggested a decreased intestinal absorption of **1**, through induction of P-gp	[67]
	Male Lewis rats; i.p. administration of **1** (1 mg/kg) beginning at the onset of the disease and then daily for 3 days, followed by injection every other day until the day of euthanization. Control rats were injected with PBS-DMSO on the same days	miRNAs (miR-22, miR-27a, miR-96, miR-142, miR-223, miR-296, miR-298 and miR-451) and their target genes in functional pathways important for RA pathogenesis miR-22, miR-27a, miR-96, miR-142, miR-223 and miR-296 were modulated by **1** Higher levels of serum miR-142, miR-155, miR-212 and miR-223 in arthritic *vs*. control rats	[68]
	Human RA FLSs; treated with **1** (50 μg/mL) for 24 h; RA–FLS1 and RA–FLS2 cells treated with **1** (0.25–2 μM) for 24 h	Impaired cell proliferation and cell cycle arrest and inhibition of RA FLS invasion Reduction of secretion of IL-6, IL-8 and MCP-1 in a dose-dependent manner; no change in the secretion of IL-10 Expression of some chemokines and chemokine receptors was altered significantly after treatment	[69]
Pristimerin (**2**)	TNF-α-stimulated human RA FLSs (MH7A cells) at 20 ng/mL and treated with **2** (0, 0.5, 1 or 2 μM) for 24 h AA male Wistar rats; intragastric administration of **2** (0.8 mg/kg/day), vehicle (0.3% CMC-Na) or MTX (0.6 mg/kg/day), for 28 days, starting the next day after CFA immunization	Inhibition of viability and migration of TNF-α-stimulated MH7A cells (IC_50_ 1.408 μM) Reduction of paw swelling, TNF-α and NO serum levels as well as p-Akt and p-ERK levels Alteration of phospholipid and fatty acid biosynthesis, glutathione metabolism and amino acid metabolic pathways Network pharmacology analysis and molecular docking studies showed that effects were mediated through the MAPK/ERK1/2, PI3K/Akt pathways and direct binding to TNF-α	[72]
Betulinic acid (**3**)	RA FLSs; pre-treatment with **3** (5, 10, and 20 μM) for 1 h and then stimulated with TNF-α (10 ng/mL) for 24 h	Inhibition of proliferation and migration of RA FLSs Attenuation of TNF-α-enhanced MMP expression in RA FLSs Inhibition of inflammatory response in RA FLSs exposed to TNF-α and prevention of the activation of Akt/NF-κB pathway	[73]
	RA FLSs treated with DMSO or **3** (0, 2.5, 5, 10 μM) for 24 h. Stimulation with TNF-α (0 or 10 ng/mL) CIA male DBA/1 mice; injected i.d. on day 0 with emulsion of BTIIC (100 mg) in CFA (1:1, *v*/*v*) and on day 21 with emulsion of BTIIC (100 mg) in IFA (1:1, *v*/*v*). CIA mice injected i.p. with **3** (20 mg/kg/day) or DMSO, for 21 days	Suppression of the migratory capacity of RA FLSs Downregulation of the mRNA expression of IL-1β, IL-6, IL-8 and IL-17A in TNF-α-induced RA FLSs Decrease in TNF-α-induced activation of NF-κB signal pathway (phosphorylated NF-κB, IκBα and IKK) and the NF-κB nuclear accumulation Inhibitory effect of NF-κB PDTC on the formation of actin stress fibers and actin cytoskeleton score of RA FLSs Attenuation of synovitis, synovial hyperplasia and invasion into calcified cartilage and bone in CIA mice	[75]
	CIA male rats twice immunized with BTIIC:CFA (1:1) injection into the right hind paw, back and tail (7 days, 2 weeks). On day 15, **3** (20 and 40 mg/kg/day, orally) or diclofenac sodium (5 mg/kg/day, orally) or ROCK inhibitor fasudil (5 mg/kg/day, i.p.) was administered for 4 weeks	Inhibition of arthritis index, amelioration of joint pathology, diminished hind paw swelling, enhanced blood rheology and synovial cell apoptosis and re-establishment of cytokine negative regulation of ROCK/NF-κB signaling pathways Decreased secretion of IL-6, IL-1β and TNF-α, inhibition of proliferation of synovial tissue, reduction of monocytes and lymphocytes Decreased levels of RhoA, ROCK1, ROCK2, p-NF-κBp65 and p-IκBα levels. Mechanistically, **3** downregulated ROCK/NF-κB signaling pathways	[74]
	CIA female albino rats; oral administration of **3** (2 mg/kg) and fluvastatin (5 mg/kg) from day 14 after arthritis induction until day 60	Decrease in the severity of arthritic index values and inhibition of paw edema on combined treatment Reduction of RF, C-reactive protein, total lipids and ACPAs; increased activity of catalase, superoxide dismutase and glutathione peroxidase enzymes and expression of the anti-inflammatory cytokine IL-10 Decreased expression of TLR2 and TLR4, IL-1β, TNF-α, IFN-γ, cell adhesion molecules and nuclear translocation of NF-κB in aorta decreased, when compared to the single-treated groups	[80]
Betulinic acid derivative SH479 (**4**)	CD4^+^ T cells and splenic lymphocytes of CIA mice treated with different concentrations Male DBA/1J mice treated with 20 mg/kg of SH479 i.p. daily beginning from day 23 after arthritis induction	*In vivo* inhibition of CD4^+^ T cell infiltration and cytokine production; inhibition of Th1 and Th17 differentiation as well as antigen-specific T cell proliferation Decrease in arthritis scores as well as bone destruction and cartilage depletion in the CIA mouse model	[82]
Lupeol (**5**)	CIA male SD rats; gastric administration of **5** (0 or 10 mg/kg) or indomethacin (3 mg/kg), from the 5th day to the 20th day after arthritic induction	Reduction of paw edema Inhibition of COX-2 and 5-LOX enzymes and reversion of the high serum levels of pro-inflammatory mediators (PGE2, TNF-α and IL-6), RF, C-reactive protein and ceruloplasmin Downregulation of Bcl-2 protein expression and upregulation of Bax, caspase-3 and -9 through PI3K/Akt inhibition	[83]
β-amyrin (**6**) and Polpunonic acid (**7**)	Sprague Dawley rats; intraplantar injection of CFA (100 μL) in AA rats and IFA (100 μL) in non-AA rats; rats treated with **6** (3, 10, 30 mg/kg, p.o.), **7** (3, 10, 30 mg/kg, p.o.) and dexamethasone (3 mg/kg, p.o.) or distilled water (10 mL/kg) once every day, for 14 days	Reduction of the primary and secondary paw swelling and the arthritis score in the later stage of the adjuvant-induced arthritis (from 14.25 to 6.5) Reversion of cartilage erosion and subchondral cyst and Weichselbaum’s lacunae formation Non-marked impact on general hematological and serum biochemical parameters due to treatment with **6**, **7** or dexamethasone	[84]
Echinocystic acid (**8**)	TNF-α-stimulated human RA FLSs at 10 ng/mL for 24 h and treated with **8** (0, 5 or 10 µM) for additional 24 h ZIA in female SKG/Jcl mice; oral administration of **8** (10 or 25 mg/kg) or vehicle (90% glyceryl trioctanoate and 10% DMSO) or MTX (10 mg/kg), i.p., daily, for 3 consecutive weeks, starting on the 21st day after single i.p. injection of zymosan A (2 mg/mice)	Reduction of synovial hyperplasia, inflammatory cell infiltration and cartilage damage on ankle joints Attenuated levels of pro-inflammatory cytokines (TNF-α, IL-6, IL-1β, IL-17A, IFN-γ and GM-CSF) and sustained reduction in joint swelling of arthritic hind paws, similar to MTX at the highest EA dose Cellular reduction of both protein and mRNA expression of IL-6 and IL-1β by downregulating MAPK and NF-κB pathways The effects were attributed to phosphorylation inhibition of STAT3 (but not JAK2) and subsequent suppression of IL-6- and TGF-β-induced Th17 cell differentiation	[85]
23-Hydroxyursolic acid (**9**)	RAW264.7 cells and primary mouse BMDMs; incubation with **9** (0, 1, 3 or 10 µM) in the presence of RANKL (100 ng/mL) and M-CSF (30 ng/mL) for 4 days (RAW264.7) or 6 days (BMDMs) LPS-stimulated ICR mice: oral administration of **9** (25 or 50 mg/kg) or vehicle (corn oil), 1 h before LPS (5 mg/kg, i.p.) injection and thereafter every other day for 8 days	Inhibition of RANKL-induced osteoclastogenesis in RAW264.7 (IC_50_ = 1.9 ± 0.2 μM) and BMDMs (IC_50_ = 2.1 ± 0.3 μM) without affecting cell viability and protected mice against LPS-induced bone loss Attenuation of osteoclast formation by inhibiting RANKL-mediated ERK and JNF phosphorylation, NF-κB signaling, c-Fos expression, NFATc1 activation and expression of osteoclast-specific marker genes (OSCAR, MMP-9, TRAP, DC-STAMP and CtsK), both *in vitro* and *in vivo*	[87]
Ursolic acid-3-acetate (**10**)	Human RA FLSs treated with **10** up to 10 μM for cell viability assays; pre-treatment with **10** for 1 h and stimulated with TNF-α for 12 h	Decrease in clinical arthritis symptoms, paw thickness, histological and radiological changes and serum IgG1 and IgG2a levels Reduction of Th1/Th17 phenotype CD4^+^ T lymphocyte expansion and inflammatory cytokine production Decreased expression and production of inflammatory mediators, in the knee joint tissue and RA synovial fibroblasts, through the downregulation of IKKα/β, ΙκBα and NF-κB	[88]
Maslinic acid (**11**)	CAIA male DBA/1J mice treated with **11** (200 mg/kg) by daily oral administration, from day 1 to day 11 Mice injected i.p. with 1 mg of a CII monoclonal antibody on day 8 and 25 μg of LPS on day 11	Lowering of arthritis score, paw thickness and front paw swelling on day 12 Suppression of the gene expression of inflammatory cytokines downstream of NF-κB signaling and inactivation of the TLR signaling pathway Downregulation of the expression levels of the genes encoding TNF-α, IL-1β, IL-6 and IL-12, and upregulation of IκBα transcript and protein expression Decrease in the production of LTB4 and alteration of the gene expression of glucocorticoids	[89]
Taraxasterol (**12**)	IL-1β-stimulated human RA FLSs pre-treated with **12** (0.3 to 30 μM), 1 h before the incubation with IL-1β (10 ng/mL) for 48 h	Downregulation of IL-1β, increase in TNF-α, IL-6, IL-8, MMP-1 and MMP-3 levels in human RA FLSs and in joint tissues of CIA mice, in a dose-dependent manner Inhibition of NF-κB activations and modulation of the TAK-1/IKK/IκB regulators in human RA FLSs and joint tissues of CIA mice, in a dose-dependent manner NLRP3, TXNIP and ASC expressions were blocked and the maturation of caspase-1 was decreased, *in vitro* and *in vivo* Reduction of clinical arthritis score and cartilage destruction in ankle joints of CIA mice Potential therapeutic action of 12 by modulation of NF-κB/NLRP3 inflammasome pathways	[90]
Wilforlide A (**13**)	LPS/IFN-γ-stimulated macrophages (THP-1 cells) treated with PMA (200 nM) for 3 days, then stimulated with LPS (1 μg/mL) and IFN-γ (100 ng/mL) and treated with **13** (0, 1, 5, 10, 20, 40, 80, 160 and 300 ng/mL) for 48 h	Reduction of inflammatory infiltration, joint swelling and histological damage in the ankle joints of CIA mice Inhibition of iNOS expression in activated macrophages of arthritic synovial joints, reduction of the high levels of pro-inflammatory cytokines (MCP1, GM-CSF and M-CSF) in joint synovium and enhanced expression of anti-inflammatory cytokines (IL-10 and TGF-β) in mouse serum *In vitro* inhibition of M1 macrophage polarization by suppressing LPS/IFN-γ-induced TLR4 upregulation, IκBα degradation and NF-κB p65 activation	[92]

### 6.2. Tetracyclic and Rearranged Triterpenes

Antcin K (**14**) is a tetracyclic ergostane-type triterpenoid isolated from *Antrodia cinnamomea*, a mushroom endemic to Taiwan and used in folk medicine due to its antioxidant, anti-inflammatory and immunomodulatory activities [93]. Antcin K (**14**) decreased pro-inflammatory cytokine production in human RA FLSs by inhibiting the phosphorylation of focal adhesion kinase (FAK), PI3K, Akt and NF-κB. Moreover, **14** also ameliorated paw swelling, cartilage degeneration and bone erosion in the CIA mouse model [93].

Ganoderic acid A (**15**), a lanostane triterpenoid extracted from *Ganoderma lucidum* (an edible mushroom), has been traditionally used in East Asia to treat inflammatory, proliferative and immunological diseases without side effects, making it a potential therapeutic agent for RA [94,95]. Cao et al. evaluated the protective effects of **15** in CIA rats to explore its therapeutic role in RA [95]. A reduction in toe swelling and arthritis index was observed, as well as an improvement in joint pathological changes and hemorheology. Serum and synovium levels of IL-1β, IL-6 and TNF-α were markedly reduced in CIA rats, and oxidative stress was regulated. **15** substantially reduced p-STAT3 and suppressor of cytokine signaling 1 (SOCS1); these results indicate a downregulation of protein expression of p-JAK3 and p-STAT3, which may lead to the regulation of the JAK/STAT signaling pathway [95]. Furthermore, protein expression levels of p-NF-κB p65 and p-IκBα in joint synovial tissue of CIA rats were reduced by **15**. The therapeutic role may also be related to the regulation of the NF-κB signaling pathway [95].

Gedunin (**16**), a limonoid-type triterpenoid isolated from several genera of the Meliaceae family, such as the Indian neem tree (*Azadirachta indica*), antagonized ROS production and reduced pro-inflammatory cytokine levels and iNOS expression in LPS-stimulated macrophages (RAW264.7 cells), TNF-α-stimulated FLSs (MH7A cells) and IL-1β-stimulated primary RA FLSs [96]. Furthermore, **16** was able to reduce paw swelling, arthritis score and cytokine production in CIA mice [96]. The *in vitro* and *in vivo* anti-inflammatory and anti-arthritic effects of **16** were due to activation of the Nrf2 signaling through inhibition of Keap1, a key oxidative stress sensor protein, by inducing p62 expression and upregulation of anti-oxidative enzymes, including heme oxygenase (HO)-1 [96].

Other studies showed that 7-deacetyl-gedunin (**17**) isolated from the fruits of *Toona sinensis* (A. Juss.) Roem suppressed ROS production and inhibited proliferation of human RA FLSs isolated from the joint synovium cave of RA patients submitted to knee surgery [97]. Compound **17** also decreased pro-inflammatory cytokine release in human FLSs (MH7A cells) but with significant inhibition of cell viability [97]. Mechanistic studies revealed that **17** exerted anti-inflammatory effects by regulating antioxidative enzymes through Nrf2 activation by inhibiting Keap1 via inducing p62 expression and antioxidant response element (ARE)-driven gene transcription [97].

Nimbolide (**18**), a major limonoid from *Azadirachta indica*, dose-dependently reduced the expression of p38 MAPK and inhibited the phosphorylation of NF-κB in IL-β-stimulated rabbit FLSs (HIG-82 cells) [98]. In a rat model of inflammatory arthritis, **18** significantly reduced STAT3 phosphorylation, attenuating STAT3 signaling with simultaneous inhibition of Notch-1 transmembrane protein receptors and NF-κB activation, thus reducing oxidative stress and pro-inflammatory cytokine levels in synovial tissue of arthritic rats [98]. Furthermore, combination therapy with both **18** (3 mg/kg/day) and MTX (2 mg/kg/week) potentiated the anti-arthritic effects of MTX while reducing its hepato-renal toxicity in a rat model of RA, presumably through antioxidant and anti-inflammatory effects [98]. The efficiency of nimbolide (**18**) was also examined by Cui et al. against joint inflammation in CIA male albino rats [99]. Treatment with **18** (20 mg/kg) resulted in a substantial increase in body weight and a pronounced reduction in arthritic index score, thymus and spleen indices, hind paw volume and edema formation, comparable to diclofenac [99]. Serum levels of IL-1β, IL-6, IL-10 and TNF-α showed a marked reduction in arthritic rats, and the activities of antioxidant enzymes were significantly improved. Supplementation with **18** downregulated the protein expression of iNOS, NF-κB, p-IκBα, IKKα and COX-2, reinforcing the contribution of nimbolide to the therapeutic strategy against RA [99].

Heilaohuacid G (**19**) is a new 3,4-seco-lanostane type triterpenoid isolated from the roots of *Kadsura coccinea*, a medicinal plant distributed in South China and used in Tujia ethnomedicine to treat RA [100]. Biological activity screening tests revealed that **19** inhibited the proliferation of RA FLSs in a concentration-dependent manner, with IC_50_ values of 8.16 ± 0.47 μM [100]. Further studies showed that **19** induced RA FLS apoptosis and suppressed inflammatory responses in LPS-induced RA FLSs and macrophages (RAW264.7 cells) by inhibiting NF-κB signaling [100].

**Table 5:** Tetracyclic and rearranged triterpenes with *in vitro*/*in vivo* RA-related effects.

Tetracyclic and Rearranged Triterpenes	Cell Model/Animal Model/Dosage	Effects and Mode of Action	Ref.
Antcin K (**14**)	Human RA FLSs (MH7A cells) treated with **14** (0, 0.3, 1, 3 or 10 μM) for 24 h CIA C57BL/6J mice treated with **14** (0, 10 or 30 mg/kg), i.p., on alternated days for 4 weeks	Inhibition of pro-inflammatory cytokines (TNF-α, IL-1β and IL-8) in human RA FLSs through downregulation of FAK, PI3K, Akt and NF-κB signaling pathways Amelioration of paw swelling, cartilage damage and bone erosion in CIA mice and decreased serum levels of TNF-α, IL-1β, IL-6 and IL-8	[93]
Ganoderic acid A (**15**)	Rats twice immunized with BTIIC:CFA (1:1) s.c. injection into the right hind paw, back and tail root (7 days, 2 weeks); on day 15, oral administration of **15** (20 and 40 mg/kg/day) or diclofenac sodium (5 mg/kg/day) or physiological saline, for 4 weeks	Improvement of glossiness, food intake and body weight of rats Reduction of swelling and limping of the hind feet, degree of toe swelling and joint inflammation Decrease in TNF-α, IL-6 and IL-1β serum and synovium levels was observed. p-JAK3, p-STAT3, SOCS1, p-NF-κB p65 and p-IκBα protein expression levels were significantly reduced The mechanism may lie in the downregulation of JAK/STAT and NF-κB signaling pathways	[95]
Gedunin (**16**)	LPS-induced macrophages (RAW264.7 cells), TNF-α-stimulated FLSs (MH7A cells) and IL-1β-stimulated primary RA FLSs; cells pre-treated with **16** (0, 1, 5, 10, 25 or 50 μM) for 1 h and incubated with 100 ng/mL LPS (RAW264.7 cells), 10 ng/mL TNF-α (MH7A cells) or 2.5 ng/mL IL-1β (RA FLSs) for 24 h CIA DBA/1 male mice; daily i.p. administration of **16** (2.5 or 5 mg/kg) or vehicle (saline, PEG400 and DMSO 6:3:1 *v*/*v*) or MTX (10 mg/kg), intragastrically, for 20 days	Reduction of iNOS expression, inhibition of IL-1β, IL-6 and TNF-α secretion and antagonization of ROS production *in vitro* Reduction of arthritis incidence, suppression of mRNA expression of IL-1β and amelioration of arthritis score, paw edema and bone erosion in CIA mice Mechanistic *in vitro* studies showed that **16** downregulated Keap1 protein expression and upregulated that of Nrf2, HO-1, NQO1 and p62, in time- and dose-dependent manners	[96]
7-Deacetyl-gedunin (**17**)	TNF-α- stimulated MH7A cells and IL-1β-stimulated human RA FLSs from the joints of RA patients; cells treated with **17** (0, 1, 2.5, 5, 10, 25, 50 75, 100 or 150 μM) for 24, 48 or 72 h after incubation with 10 ng/mL TNF-α (MH7A cells) or 2.5 ng/mL IL-1β (RA FLSs)	Suppressed cell proliferation, inhibited ROS production and downregulated MMP-1, -3, -9 and -13 without cytotoxicity (IL-1β-treated cells) Downregulation of IL-6 and IL-33 with inhibition of cell viability (TNF-α-treated cells) Mechanistically, **17** increased the expression of anti-oxidative enzymes (HO-1 and NQO1) and p62, thus downregulating Keap1 and activating Nrf2	[97]
Nimbolide (**18**)	IL-1β stimulated rabbit FLSs (HIG-82 cells) pre-treated with **18** (0, 0.5 or 1 μM) for 24 h and stimulated with IL-1β (10 ng/mL) for next 6 h AA Wistar rats injected with CFA (100 μL, i.a.) in the knee joint and treated with **18** (1 or 3 mg/kg) or vehicle (1% DMSO), i.p., daily, for 21 days	Inhibition of the migration of FLSs *in vitro* (IC_50_ 3.29 ± 0.15 μM) and decreased expression levels of iNOS, COX-2, MMP-2 and p38. Suppressed nitroso-oxidative stress and reduced the levels of iNOS, COX-2, IL-6 and MMP-2, both *in vitro* and *in vivo* Decrease in synovial hyperplasia, prevention of cartilage destruction, pain attenuation and amelioration of arthritis progression *in vivo* by abrogating the STAT3/NF-κB/Notch-1 signaling pathway in synovial tissue of arthritic rats	[98]
	Rats injected with CFA (100 μL, i.d.) into the right hind footpad and treated with vehicle (DMSO), **18** (20 mg/kg/day) or diclofenac sodium (5 mg/kg/day), by oral gavage, for 25 days	Significant body weight increase Decrease in paw volume and arthritic index score and in activities of liver marker serum enzymes (SGOT, SGPT, ALP) Reduction of serum levels of TNF-α, IL-6, IL-1β and IL-10. Decrease in MDA levels and enhancement in activities of antioxidant enzymes; outcomes were comparable to diclofenac sodium **18** reduced the higher protein levels of COX-2, iNOS, NF-κB, p-IκBα and IKKα in CFA-induced RA rats	[99]
Heilaohuacid G (**19**)	LPS-stimulated human RA FLSs and macrophages (RAW264.7 cells). Cells treated with **19** (0, 2.5, 5, 10 or 20 μM) for 24 h (RAW264.7) or 48 h (RA FLSs) and then incubated with LPS (100 ng/mL) for another 4 h	Inhibition of RA FLS proliferation with IC_50_ value of 8.16 ± 0.47 μM Induction of RA FLS apoptosis and inhibition of the secretion of pro-inflammatory cytokines Reduced TNF-α and IL-6 in LPS-induced RA FLSs and RAW264.7 cells by suppressing NF-κB signaling	[100]

### 6.3. Triterpenic Saponins

Astragaloside (**20**), a saponin found in *Astragalus membranaceus*, suppressed excessive FLS proliferation in the AA rat model of RA through the inhibition of the expression of the long non-coding RNA (lncRNA) LOC100912373 and increased release of miR-17-5p, which binds to 3-phosphoinositide-dependent protein kinase 1 (PDK1) and prevents activation of the PDK1/Akt pathway [101]. Abnormal expression of non-coding RNAs, such as miRNAs and lncRNAs, has been implicated in the pathogenesis of autoimmune diseases, including RA [102]. lncRNAs are expressed by many immune system cells, including T and B lymphocytes, monocytes, macrophages and dendritic cells, and lncRNA dysregulation has been associated with autoimmunity onset [102]. Additionally, lncRNAs can act as molecular sponges, sequestering miRNAs and RNA-binding proteins, hindering interactions with their target RNAs [102]. The lncRNA LOC100912373 is a critical gene involved in RA pathogenesis since it can induce FLS proliferation by competing with miR-17-5p and thus promoting activation of the PDK1/Akt signaling pathway that contributes to RA development [103].

Chikusetsusaponin IVa (**21**), an oleanane-type saponin from *Panax japonicus* C.A. Mey, alleviated RA symptoms in CIA mice [104]. Molecular docking and molecular dynamics simulations revealed that **21** can bind to RA core targets IFN-γ and IL-1β. The study results suggest that the *in vivo* anti-inflammatory and osteoprotective effects of **21** were due to inhibition of the JAK/STAT signaling pathway [104].

Immunopathology in RA is driven by a predominance of arthritogenic Th1 cells (secreting IFN-γ) and Th17 cells (secreting IL-17) over T_reg_ cells [105]. Cytokine IL-6 is critical for the differentiation of Th17 cells and the balance between pathogenic Th17 and protective T_reg_ [105]. Biologic DMARDs targeting the IL-6 receptor have been shown to improve signs and symptoms of RA. Chikusetsusaponin IVa butyl ester (**22**) is a triterpenoid saponin extracted from *Acanthopanas gracilistylus* and a small-molecule IL-6R inhibitor. IL-6R blockade by **22** inhibited Th17 cell differentiation, IL-17A secretion and STAT3 phosphorylation in mouse CD4^+^ cells (under Th17 polarization conditions) *in vitro* and ameliorated RA symptoms in the CIA mouse model [106]. Thus, saponin **22** represents a promising agent for RA therapy.

Circular RNAs (circRNAs), which are endogenous non-coding RNAs forming stable covalently closed-loop structures, act as miRNA sponges and participate in the regulation of several cellular signaling pathways [102,107]. circRNAs are important epigenetic modulators of gene expression in inflammation and autoimmune regulation, closely associated with RA pathogenesis [102,107]. Clematichinenoside AR (**23**) is a triterpenoid saponin isolated from the roots of *Clematis chinensis* Osbeck. Saponin **23** inhibited proliferation and inflammatory response in FLSs from RA patients *in vitro* and ameliorated RA pathology in CIA mice by combining with frizzled class receptor 4 (FZD4) and blocking the circular pleiotrophin (circPTN)/miR-145-5p/FZD4 signal axis [108]. The authors demonstrated that circPTN promoted FZD4 expression through sponging miR-145-5p with subsequent activation of the Wnt/β-catenin pathway [108]. Confocal microscopy showed that **23** downregulated the expression of β-catenin and its nuclear entry in FLSs by binding FZD4, thus inhibiting the Wnt pathway [108]. Compound (**23**) was also the focus of Xiong et al., who explored its protective action against human TNF-α-induced inflammation and cytotoxicity based on the accumulated evidence about the correlation between RA therapeutic effects and the antagonist effects against TNF-α in RA mouse models [109]. **23** markedly inhibited IL-6 and IL-8 release from recombinant human (rh) TNF-α-stimulated MH7A cells. Cartilage and bone destruction were reversed, probably through downregulation of MMP-1 expression and downregulation of p38 and ERK MAPK signal activation by **23** in rhTNF-α-induced MH7A cells [109]. Treatment of TNF-α-sensitive murine fibroblast L929 cells with **23** reduced the proliferation inhibition ratio caused by rhTNF-α/actinomycin D (ActD) and antagonized rhTNF-α-induced cytotoxicity. Morphological changes in apoptosis (including chromatin condensation, nuclear fragmentation and cell shrinkage) stimulated by rhTNF-α/ActD in L929 cells were attenuated after pre-treatment with **23** [109]. The antagonistic effect of **23** upon cytotoxicity might be ascribed to the degeneration of ROS and the raising of mitochondrial membrane potential, together with the inhibition of prolonged JNK activation following pre-treatment.

Entadaosides **24**–**28**, oleanane-type triterpene saponins isolated from the stems of *Entada phaseolides* (L.) Merr, possess anti-inflammatory properties and are used in traditional Chinese medicine for the treatment of arthritis [110]. All entadaoside saponins **24**–**28** were able to prevent RA progression and ameliorate hyperalgesia, paw swelling and joint destruction in CIA rats by reducing pro-inflammatory cytokine levels, upregulating ubiquitin-editing enzyme A20 expression, inhibiting p38 and ERK1/2 in the periphery and phosphorylation of p38 in the spinal cord [110].

Madecassoside (**29**) is a pentacyclic triterpenoid saponin present in *Centella asiatica*, with previously reported anti-inflammatory and anti-arthritis potential, among other important biological activities. It was also found to induce apoptosis of keloid fibroblasts and keratinocytes and to inhibit LPS-induced TNF-α production, as well as the migration of keloid fibroblasts [111]. Yu et al. used IL-1β stimulation to induce the invasion of FLSs, aiming at exploring the anti-arthritis mechanism of saponin **29** [112]. It was found that oral administration of the triterpenoid exerted a significant therapeutic effect, reducing the articular and bone tissue damage and decreasing hyperemia in the synovial tissue. A dose-dependent *in vitro* inhibitory effect on FLS invasion mediated by IL-1β was also observed, as well as a decrease in MMP-13 activity and mRNA level expression, possibly by preventing NF-κB translocation and phosphorylation.

Qiao et al. compared the anti-arthritis effect of madecassoside (**29**) and its metabolite madecassic acid in pseudo-germ-free CIA rats, discussing the influence of gut microbiota and the mechanism of **29** to stimulate T_reg_ cells [113]. Previous studies revealed the potential of **29** to increase the number of T_reg_ cells in the small intestine, improving the release of IL-10 through the increase in Foxp3^+^ T lymphocytes in the intestinal lamina propria. However, neither **29** nor its metabolite was able to foment the differentiation of T_reg_ cells and the expression of IL-10 in CD4^+^T cells of CIA rats [113]. In the comparison study, oral administration of **29** was shown to mitigate the arthritis symptoms and the histological alterations in CIA rats, unlike intestinal madecassic acid, suggesting its functionality in the parent form. The increased number of T_reg_ cells by oral administration of **29** was observed mainly in the ileum but without a significant effect concerning T_reg_ cell differentiation and Foxp3 and IL-10 expression *in vitro*. The anti-arthritis effect of compound **29** was strongly influenced by gut microbiota; the sequencing of the 16S rRNA gene indicated that **29** antagonized the richness and diversity of gut microbiota in CIA rats, enhancing the level of n-butyric acid (which increased the immunosuppressive function of T_reg_ cells *in vitro*). The co-administration of heptanoyl CoA (a competitive inhibitor of butyrate synthase) confirmed the contribution of madecassoside-induced butyrate to the anti-arthritis action, as it caused the downregulation of ileum T_reg_ cell number and expression of Foxp3 and IL-10 [113].

Mussaendoside O (**30**), a *N*-triterpene cycloartane saponin isolated from *Mussaenda pubescens*, inhibits RANKL-induced osteoclastogenesis *in vitro* in a concentration-dependent manner [114]. Moreover, 30 attenuates LPS-induced bone resorption and osteoclast formation in mice by repressing RANKL-induced activation of p38 MAPK and JNK, preventing c-Fos activation and subsequent expression of NFATc1. Saponin **30** also diminished RANKL-induced increase in mRNA expression of NFATc1 target genes, including OSCAR, TRAP, DC-STAMP and cathepsin K [114].

Tubeimoside I (**31**) is a triterpenoid saponin previously isolated from *Bolbostemma paniculatum* tubers and found in several Chinese medicine preparations, with anti-inflammatory, anti-tumor and anti-viral activities [115]. The effect of this compound on RA was studied *in vivo* using a CIA rat model and *in vitro* using cultured FLSs [116]. The treatment with **31** suppressed the synovial inflammation and bone destruction in CIA rats in a dose-dependent manner, decreasing erythema and swelling at the doses of 5 and 10 mg/kg. These results were further confirmed by histopathological assays. Moreover, when compared with the control group, an important decrease in pro-inflammatory cytokine production was observed in the joint tissues of tubeimoside I-treated rats, including IL-1β, IL-6, IL-8 and TNF-α, and downregulation of MMP-9 expression. *In vitro* studies also showed that the compound suppressed the proliferation and migration of FLS cells, which are the main causes of synovial hyperplasia, contributing to the cartilage destruction and exacerbating joint damage. The authors suggested that the observed effects may be due to the inhibition of TNF-α-induced activation of NF-κB and MAPKs (p38 and JNK) [116].

Ginsenosides are glycosylated dammarane-type triterpenoids unique to ginseng species. Ginseng is a drug derived from the roots of *Panax ginseng*, used in traditional medicine to treat several diseases, including anemia, diabetes, gastritis and insomnia. It is also used as a general restorative, promoting health and longevity [21]. Based on the location and number of glycoside residues, ginsenosides can be further subdivided into protopanaxadiols (e.g., Rb1, Rb2, Rg3, Rg5 and Rh2), with glycoside residues attached to C-3 and/or C-20 positions, or protopanaxatriols (e.g., Rg1, Rg2 and Rh1), with an additional glycoside residue at C-6 (Figure 4).

Zhang et al. compared several ginsenosides (CK, Rg1, Rg3, Rg5 and Rb1; **32**–**36**) from *Panax ginseng* Meyer for their therapeutic effect on RA [117]. Ginsenoside CK (**32**) is the major metabolite of natural diol ginsenosides in the intestinal tract [117]. Among the tested ginsenosides, **32** was the most effective, showing strong anti-inflammatory and immunomodulating properties [117]. Ginsenoside CK (**32**) significantly inhibited cell proliferation and enhanced apoptosis of LPS-activated RAW264.7 and TNF-α-stimulated human umbilical vein endothelial cells (HUVECs) [117]. *In vivo*, **32** ameliorated swelling and joint functional impairment in CIA mice [117]. Moreover, **32** was able to increase CD8^+^ T cells to downregulate the immune response and decrease the number of activated CD4^+^ T cells and M1 macrophages, thus inhibiting pro-inflammatory cytokine secretion [117]. In an attempt to explore the mechanism of macrophage polarization and phagocytosis by compound **32**, Wang et al. concluded that, through β-arrestin2 regulation in peritoneal macrophages, the compound inhibited TLR4 coupling with Gαi and stimulated TLR4-Gαs coupling [118]. Due to the significant decrease in colocalization of β-arrestin2 and Gαi, owing to **32** treatment, their combined interaction foments the regulation of immune inflammation and the polarization of macrophages to M1. Potential therapeutic properties of **32** for RA therapy seem to be related to the reduction of M1 polarization and secretion of inflammatory cytokines, while overexpressing M2 and IL-10 levels to alleviate inflammation and repair bone tissue in CIA mice. Compound **32** also appears to restore B cell function, in addition to alleviating clinical manifestations of RA (such as the polyarthritis index, spleen and joint pathological scores and spleen index) and the level of serum antibodies in CIA mice [119]. Although IgD B cell receptor (BCR) endocytosis was promoted, it should be noted that the expression level of IgD-BCR did not change. **32** facilitated the co-localization between β-arrestin1 and IgD and between adaptor protein 2 (AP2) and IgD. Mechanistically, the IgD-BCR internalization, in a β-arrestin-AP2-dependent manner, led to the inhibition of B cell activation, which may explain the improvement observed in CIA model mice [119].

Ginsenoside CK (**32**) is thus a potential candidate for RA therapy and is currently being tested as an anti-RA drug in China. Phase 1 clinical trials in healthy Chinese volunteers to evaluate the pharmacokinetics and safety of **32** showed that a single oral dose of a 200 mg tablet was well tolerated, reaching a maximum plasma concentration (*C*_max_) of 796.8 ng/mL in 3.6 h (*T*_max_) with a terminal half-life (*t*_1/2_) of 27.7 h [120]. High-fat food was found to accelerate and increase absorption of **32** while plasma levels were slightly higher in women compared to men [120]. A double-blind, phase 2 study (NCT03755258) to evaluate the safety, efficacy and pharmacokinetics of ginsenoside CK (**32**) tablets in RA patients started in China in March 2017. RA patients (*n* = 128) were randomly assigned ginsenoside CK tablets (100, 200 or 300 mg) or placebo once daily, orally, for 12 weeks. However, the study was suspended after two years due to the high cost associated with manufacturing of **32**, essentially dependent on *Panax* plants, extraction and biotransformation of ginsenosides. Therefore, development of alternative production methods, such as microbial fermentation processes suitable for scale-up, is an attractive solution.

Many studies on the anti-inflammatory effect of ginsenoside Rg3 (**34**) have been described, emphasizing its ability to regulate NF-κB activity, causing the reduction of cytokine levels, to promote M2 macrophage polarization and to inhibit the inflammation process in the liver through the activation of the PI3K/AKT signaling pathway [53]. Considering the lack of mechanistic robustness regarding the effect of ginsenoside Rg3 in RA, Zhang et al. evaluated the anti-inflammatory effect of the compound **34** through a set of clinical features, pathological alterations and cytokine levels observed in RA mice. CD4^+^CD25^+^Foxp3^+^ T_reg_ cell percentage was analyzed and a metabolomic analysis (GC-MS/MS) was performed, aiming to provide information on immunosuppressive activity and related mechanisms [53]. Treatment with **34** (25 mg/kg) led to a decrease in IL-6 and TNF-α levels and an increase in TGF-β and IL-10 levels, mirroring its anti-inflammatory potential. **34** regulated the pathways of oxidative phosphorylation and maintained peripheral immune tolerance in RA mice, enhancing the function of CD4^+^CD25^+^Foxp3^+^ T_reg_ cells [53].

**Table 6:** Triterpenic saponins with in vitro/in vivo RA-related effects.

Triterpenic Saponins	Cell Model/Animal Model/Dosage	Effects and Mode of Action	Ref.
Astragaloside (**20**)	AA rat FLSs incubated with **20** (0, 7.8, 15.6, 31.25, 62.5, 125, 250 or 500 mg/L) for 24, 48 or 72 h at 37 °C AA in male SD SPF grade rats; rats were immunized with single CFA injection into the left hind foot and studied for 20 days	Inhibition of FLS proliferation, reduction of lncRNA LOC100912373 expression, increased miR-17-5p expression and decreased PDK1 and p-AKT levels Reversion of the effects of LOC100912373 overexpression on FLS proliferation and cell cycle progression by regulating the expression of LOC100912373 and the miR-17-5p/PDK1 axis	[101]
Chikusetsusaponin IVa (**21**)	CIA in DBA/1J mice; treatment with **21** (50 or 100 mg/kg), dexamethasone (0.2 mg/kg) or saline (negative control) with additional treatments between days 28 and 40	Reduction of arthritis index, joint synovial inflammation, paw edema and bone loss in CIA mice Decrease in both rat serum levels and mRNA expression of inflammatory cytokines (IL-1β, IL-6, IFN-γ and TNF-α) and inhibition of protein expression levels of JAK1, JAK2, STAT3 and p-STAT3 in the rat synovial tissue Molecular docking and molecular dynamics simulations revealed that **21** binds to IFN-γ and IL-1β	[104]
Chikusetsusaponin IVa butyl ester (**22**)	Naïve CD4^+^ cells from C57BL/6 mouse spleens, incubated with **22** (0, 2.5, 5.0, 7.5 or 10 μM) for 24 h before stimulation under Th17 polarizing conditions for 3 days CIA DBA/1J mice treated with **22** (20 or 40 mg/kg) or vehicle (5% Solutol^®^ HS), 6 days weekly for 8 weeks from first immunization	Decrease in arthritis scores, inflammation scores of the ankle joints, hind paw swelling and ankle joint bone erosion in CIA mice Reduction of Th17 cells and increased T_reg_ cells, reversing the abnormal Th17/T_reg_ ratio in the spleens of CIA mice *In vitro* inhibition of Th17 cell differentiation, IL-17A secretion and STAT3 phosphorylation and decrease in mRNA levels of IRF4 and RORγT in splenic CD4^+^ cells under Th17 polarization conditions	[106]
Clematichinenoside AR (**23**)	FLSs from RA patients and CIA mice incubated with **23** (0.187 mg/L) for 36 h CIA DBA/1 SPF grade mice administered **23** (0, 0.18, 0.37, 0.75 or 1.5 mg/kg) or MTX (0.75 mg/kg), by oral gavage, on the 28th day after first immunization	Inhibition of the arthritis score of CIA mice, reduction of paw swelling and restored mouse body weight Suppression of FLS proliferation, secretion inhibition of IL-1β, IL-6 and IL-8 and reduction of the expression of β-catenin, fibronectin and MMP-3 *in vitro* Inhibition of the Wnt/β-catenin pathway by binding to FZD4 and blocking the circPTN/miR-145-5p/FZD4 signal axis	[108]
	Human RA FLSs (MH7A cells) incubated with **23** (3, 10 or 30 μM) for 1 h, followed by exposure to rhTNF-α (10 ng/mL) for 24 h TNF-α-sensitive mouse fibroblast (L929) cells pre-incubated for 1 h with **23** (1 10 or 100 μM), followed by rhTNF-α (5 ng/mL) stimulation in the presence of ActD (0.5 μg/mL) for 24 h	Significant reduction of IL-6 secretion and IL-8 production in a concentration-dependent mode, in MH7A cells stimulated by recombinant human TNF-α Decrease in rhTNF-α-induced MMP-1. Suppression of phosphorylated levels of p38 and ERK1/2 produced by rhTNF-α. Abolition of rhTNF-α-induced L929 cell cytotoxicity Attenuation of L929 cells’ morphological induced modifications (increase in cell density and decrease in apoptotic morphology levels) Mechanistically, anti-destructive effects of **23** caused by rhTNF-α may be through the downregulation of MMP-1 expression, and the protective effects of murine L929 cells may lie in the suppression of JNK continuous phosphorylation	[109]
Entadaosides (**24**–**28**)	CIA Wistar rats treated by oral gavage with each entadaoside (25, 50 or 100 mg/kg/day), celecoxib (18 mg/kg/day) or saline (negative control) for 3 weeks	Reduction of mRNA levels and production of pro-inflammatory cytokines (TNF-α, IL-17) in synovial tissues and hind paw joint Upregulation of A20 and inhibition of ERK1/2 activation in hind paw joints as well as p38, both in the periphery and spinal cord	[110]
Madecassoside (**29**)	CIA-induced Wistar rats twice injected i.d. at the base of tail with emulsion CII in CFA (1 mg/mL), on day 0 (200 μL) and day 7 (100 μL). From day 14 to day 30, oral administration of **29** (30 mg/kg) or madecassic acid (15 mg/kg) or vehicle (CMC-Na). Co-administration of heptanoyl CoA (0.3 mg/kg) with MAD, through insertion of a Teflon canula into the anus (8 cm), from day 14 to day 34.	Decrease in the maximum paw swelling and arthritis index score; improvement of body weight loss and histological changes (joints’ synovial hyperplasia, inflammatory cell infiltration and cartilage and bone destruction) Reversion of changes in gut microbiota, rise in acetic acid and butyric acid levels Selective promotion of the production of T_reg_ cells in the parent form (**29**), although *in vitro* the effects on T_reg_ cell differentiation and the expression of Foxp3 and IL-10 were not so significant Increase in the expression of T_reg_ cells and promotion of the expression of Foxp3 and IL-10 in rat ileum (rather than duodenum and jejunum), fomented by sodium butyrate (in a concentration-dependent mode)	[113]
	AIA rat model treated by oral gavage with **29** (25 mg/kg) and dexamethasone (positive control, 0.5 mg/kg) for 13 days	Inhibition of migration and invasion of FLSs induced by IL-1β; suppression of IL-1β-triggered FLS invasion through suppression of MMP-13 activity and transcription via inhibiting the MMP-13 promoter-binding activity of NF-κB and downregulating the translocation and phosphorylation of NF-κB	[112]
Mussaendoside O (**30**)	Mouse BMDMs and RAW264.7 cells incubated with **30** (0, 0.3, 1 or 3 μM) in the presence of RANKL (100 ng/mL) and M-CSF (30 ng/mL) for 4 days (RAW264.7) or 7 days (BMDMs) LPS-stimulated ICR mice treated with **30** (10 or 20 mg/kg) or vehicle (corn oil), orally, 1 h before the first injection of LPS (5 mg/kg, i.p.) and thereafter every other day for 8 days	Inhibition of RANKL-induced osteoclast differentiation in BMDMs (IC_50_ 0.75 ± 0.15 μM) and RAW264.7 (IC_50_ 0.75 ± 0.15 μM), without decreasing cell viability **30** failed to inhibit LPS-induced production of pro-inflammatory mediators (NO, iNOS, COX-2 and TNF-α) in RAW264.7 cells Inhibition of RANKL-induced osteoclastogenesis *in vitro* was attributed to the impairing of c-Fos and subsequent NFATc1 expression At 20 mg/kg, **30** significantly protected mice against LPS-induced bone loss presumably by suppressing c-Fos expression through inhibition of JNK and p38 MAPK pathways	[114]
Tubeimoside I (**31**)	CIA Wistar rats; i.p. administration of **31** (1, 5 or 10 mg/kg/day)	Synovial inflammation and bone destruction were suppressed in CIA rats in a dose-dependent manner Decrease in pro-inflammatory cytokine production	[116]
Ginsenosides CK, Rg1, Rg3, Rg5 and Rb1 (**32**–**36**)	LPS-activated RAW264.7 cells and TNF-α-stimulated HUVECs; cells treated with 100 ng/mL LPS (RAW264.7) or 10 ng/mL TNF-α (HUVECs) for 24 h followed by incubation with each ginsenoside (1.5625, 3.125, 6.25, 12.5, 25, 50, 100 or 200 μg/mL), MTX (positive control) or DMSO (negative control) CIA male DBA/1 mice treated with 15 mg/kg ginsenosides (**32**–**36**) or vehicle (0.5% Tween-80), i.v., once every 2 days, 15 times, after onset of joint swelling	All ginsenosides **32**–**36** showed good therapeutic effect on acute arthritis. Among the tested ginsenosides, **32** was the most effective *In vitro*, **32** inhibited cell proliferation and enhanced apoptosis *In vivo*, **32** reduced swelling and joint functional impairment in CIA mice **32** increased CD8^+^ T cells to downregulate the immune response and decreased the number of activated CD4^+^ T cells and pro-inflammatory M1 macrophages, inhibiting the secretion of TNF-α and IL-6	[117]
Ginsenoside CK (**32**)	CIA DBA/1 mice; CIA induced by two i.d. injections in the tail root with 100 μL emulsion of CII (1 mg/mL) and Calmette’s vaccine (2 mg/mL), on days 0 and 21. On day 28, mice treated with intragastric administration of **32** (112 mg/kg/day) or MTX (2 mg/kg/day), for 24 days.	**32** restored mouse body weight and alleviated symptoms of arthritis Spleen index was attenuated, and proliferation of splenic and thymic lymphocytes was inhibited Secretion of IL-1β, IL-17 and TNF-α was decreased. IL-10 level was promoted in serum and macrophage culture supernatants. M1 and M2 macrophages were diminished and augmented, respectively Inhibition of the expression of Gαi, TLR4 and NF-κB, increasing Gαs level. The performance of **32** was similar to MTX. But unlike MTX, **32** inhibited the expression of β-arrestin2. Through β-arrestin2 regulation in macrophages, **32** inhibited TLR4–GαI coupling and promoted TLR4–Gαs coupling	[121]
	CIA male DBA/1 mice; CIA induced by two i.d. injections in the tail root with 100 μL emulsion of CFA and CCII (at equal volumes), on days 0 and 21. Mice treated with **32** (28, 56 or 112 mg/kg) or MTX (2 mg/kg) or vehicle (CMC-Na), from day 28 to day 51	Improvement of the polyarthritis index, swollen joint count, spleen and joint pathological scores and spleen index Abnormal B cell spreading was inhibited. Production of serum antibodies (IgG1, IgG2a, anti-CII) was prevented, and the pathogenesis of CIA was improved. These outcomes were more pronounced with **32** (112 mg/kg) and in a similar trend to MTX Homeostasis of B cell subsets (regulatory B cells, plasma cells, memory B cells, mature B and FO B cells) was restored in CIA mice **32** promoted co-localizations between IgD and β-arrestin1 and between IgD and AP2. Although **32** did not alter IgD-BCR expression, it seemed to foment IgD-BCR internalization in a β-arrestin1-AP2-dependent manner	[119]
Ginsenoside Rg3 (**34**)	Mice immunized with a single s.c. injection of CFA (100 μL) into the right hind footpad. On day 7, mice treated intragastrically with saline (100 μL) or **34** (25 mg/kg/day) for 16 days	**34** reduced the swelling rates of RA mice, decreased the degree of cartilage destruction and vasodilation, diminished protein expression of TNF-α and IL-6 and raised the protein expression of IL-10 and TGF-β in the ankle joint Enhancement of oxidative phosphorylation and reinforcement of the TCA cycle and the respiration of ETC. CD4^+^CD25^+^Foxp3^+^ T_reg_ cell percentage was increased; lipids played a crucial role in the proliferation and differentiation of these cells	[53]

## 7. Conclusions and Future Perspectives

Morbidity and mortality associated with RA justify the continuous interest in the quest for new compounds with better efficacy and a mechanistic rationale, together with a deeper understanding of the anti-arthritis effects of novel isolated compounds or those already reported as therapeutic compounds in RA. Being a systemic autoimmune and chronic inflammatory disease, RA displays a significant increase in macrophages, chemokines, inflammation cytokines, B cells, CD4^+^ T cells and autoantibodies. Current diagnostic biomarkers for RA consist of ACPAs and less specific RFs, along with synovial inflammation, cartilage and bone destruction and systemic disorders. Under inflammatory conditions, FLSs are implicated in the production of pro-inflammatory cytokines and chemokines, extracellular matrix-degrading enzymes and pro-angiogenic factors. These pro-inflammatory cytokines and chemokines include IL-1, IL-2, IL-3, IL-4, IL-6, IL-8, IL-17, IL-18, IFN-α and IFN-β, TNF-α, TGF-β, GM-CSF and macrophage inflammatory protein (MIP)-3α. The synovial inflammation results from the activation of NF-κB, production of PGE2 and upregulation of COX-2 expression, all of which are promoted by pro-inflammatory cytokines and chemokines. TLR signaling and the NLRP3 inflammasome also appear to have potential roles in the pathogenesis of RA. Current pharmacological approaches to managing RA involve DMARDs alone or in combination with NSAIDs or low-dose glucocorticoids. However, probable and considerable toxicity related to DMARDs affects their ability to treat the disease, fomenting the need to find new therapeutic options.

The use of medicinal plants to treat RA has a long-established tradition of efficacy. Yet, few types of natural medicines for RA treatment are available, most of which are involved in pre-clinical research. Celastrol, the principal active constituent of *Tripterygium wilfordii*, has been demonstrating great therapeutic potential in the treatment of RA, although its mechanism of action is far from being established. Nevertheless, its toxicity is a troublesome issue, affecting the gastrointestinal tract, liver and reproductive system. Increasing the number of bioactive compounds, with high quality and low toxicity, and identifying the mechanism(s) of action of plant components are of extreme importance, while also exploring new potential medicinal herbs. Natural compounds may be valuable alternative choices for drugs in RA treatment, either as adjuvants to conventional drugs or as therapeutic agents.

Triterpenes are a large and structurally diverse group of natural compounds with documented anti-inflammatory and immunomodulatory activities. In this review, thirty-six triterpenoids were divided into pentacyclic triterpenes, tetracyclic and rearranged triterpenes and triterpenic saponins and examined regarding their potential effects on RA, as they target and revert a large number of signaling pathways and cytokines, both *in vitro* and *in vivo* in several animal models of RA. 

Anti-RA effects of the described triterpenoids mainly rely on their known anti-oxidative and anti-inflammatory properties. Triterpenoids can reduce oxidative stress by enhancing superoxide dismutase activity, inhibiting NADPH oxidase activity and decreasing malondialdehyde and superoxide anions levels [70,80]. The reported upregulation of anti-oxidative enzymes by triterpenoids has been attributed to activation of the Nrf2/ARE signaling pathway [96,97]. Triterpenoids are also capable of inhibiting COX-2 and 5-LOX enzymes [83], thus inhibiting the biosynthesis of prostaglandins and leukotrienes, respectively, which are important mediators of the inflammation process. 

**Figure 6:**
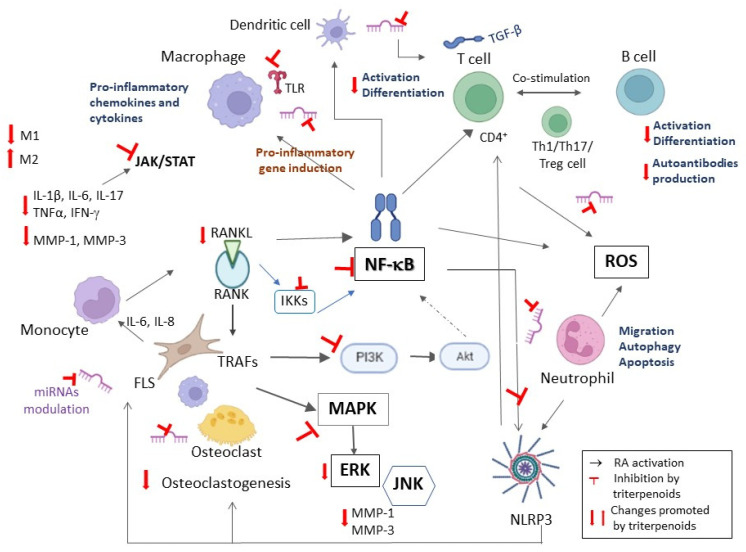
Main modes of action of anti-RA triterpenoids. Created with BioRender.com (accessed on 14 June 2023).

Among the different modes of action that have been described for anti-arthritic triterpenoids (Figure 6), inhibition of NF-κB signaling is the major one [63,74,75,80,85,87,88,90,92,95,98,99,100,112,116]. Deregulated NF-κB activation is characteristic of chronic inflammatory diseases, such as RA [122]. The transcription factor NF-κB is known to play a pivotal role in the regulation of both innate and adaptive immune responses and it is a key mediator of the inflammatory process. NF-κB can induce the expression of several pro-inflammatory genes, including those encoding pro-inflammatory cytokines and chemokines, and is involved in the activation and differentiation of innate immune cells and inflammatory T cells [122]. Furthermore, triterpenoid inhibition of RANKL-induced osteoclastogenesis strongly contributes to the prevention of bone damage and disease progression in animal models of RA [87,114].

Other modes of action of anti-arthritic triterpenoids include inhibition of PI3K/Akt [53,65,72,83,93] and MAPK/ERK [72,85,109,110,116] signaling pathways, hindering of NLRP3 inflammasome activation [63,90] and modulation of miRNAs and their target genes involved in functional pathways relevant for RA pathogenesis [68]. Triterpenoid inactivation of TLR signaling [80,89,92,118], which hinders macrophage chemotaxis and M1 polarization, is another mechanism responsible for the anti-arthritic effects of this class of compounds. Suppression of both protein and mRNA expression of pro-inflammatory cytokines, such as IL-6 and IL-1β, through inhibition of the JAK/STAT signaling pathway has also been reported [85,95,98,99,104,106], with subsequent suppression of IL-6 and TGF-β-induced Th17 differentiation. The production of TNF-α-induced pro-inflammatory cytokines (IL-6 and IL-8) has also been inhibited by direct binding of the triterpenoid to TNF-α [72].

Additionally, these bioactive triterpenoids were able, in general, to produce a reduction in several RA activity indices, including paw edema, arthritis scores, body weight and hematological, biochemical and immunological markers. In the considered timespan of this review, pentacyclic triterpenes from *Tripterygium wilfordii*, such as celastrol, and betulinic acid, stand out as the most studied compounds with a deep investigation of their molecular mechanism. Nimbolide, a limonoid triterpene, has also been considered a potential therapeutic strategy against RA, and its contribution has been well addressed. Several ginsenoside compounds have been described as being effective in the treatment of RA, with ginsenoside CK appearing to have stronger anti-inflammation and immunomodulatory properties among them.

This review highlights the significant progress in the research concerning triterpenoids as potential agents in the management of RA. In the future, continued contributions from basic research, more comprehensive and in-depth research and well-controlled clinical trials are required. Knowledge gaps in triterpene mechanisms of action need to be addressed in future research. Cell and serum metabolomics profiling of the effects of some of the above triterpenoids has already been successfully established, paving the way for analytical profiling approaches such as metabolomics, proteomics or transcriptomics to provide mechanistic clarifications. Since gut microbiota plays a crucial role in health and disease, and some triterpenoids were shown to be affected by gut microbiome composition, this field could be further explored. Despite their numerous and/or potential pharmacological properties in the treatment of RA, triterpenoids show low bioavailability and toxicity. Toxicity evaluations have been lacking, which is expected to be handled in the future. On the other hand, investing more in the development of targeted drug delivery systems containing triterpenoids could overcome these significant drawbacks. 

## Figures and Tables

**Table 1:** Global prevalence, incidence and years lived with disability (YLDs) attributable to RA for men, women and both genders in 2019 with percentage change (numbers in parentheses) between 2010 and 2019. Data from Global Burden of Disease Collaborative Network, 2020 [25].

Gender	Prevalence Cases (Millions)	Incidence Cases (Millions)	YLDs Counts (Millions)
Male	5.39 (23.9%)	0.330 (20.1%)	0.716 (23.3%)
Female	13.2 (21.7%)	0.744 (17.3%)	1.72 (21.2%)
Overall	18.6 (22.3%)	1.07 (18.1%)	2.43 (21.8%)

**Table 2:** Summary of symptoms, risk factors and common comorbidities of RA.

**Commonly affected joints**	Hands, wrists, knees and feet, typically in symmetrical pattern.
**Symptoms**	Pain, tenderness, early morning stiffness lasting 30 min or longer and swelling involving multiple (peripheral) joints bilaterally, low-grade fever, fatigue and weight loss.
**Main risk factors**	Advancing age, female sex, positive family history/genetics, overweight/obesity, smoking, particulate matter exposure, infectious agents, microbiome dysbiosis, stress and pro-inflammatory diet (rich in fried foods, processed foods, refined carbohydrates, sodas and red meat).
**Common comorbidities**	Cardiovascular disease, lymphoma, interstitial lung disease, pulmonary fibrosis, vasculitis, metabolic syndrome, type 2 diabetes, atherosclerosis, osteoporosis, anemia, dry keratoconjunctivitis and depression.

**Table 3:** Major classes of disease-modifying anti-rheumatic drugs (DMARDs) currently in the market.

Synthetic DMARDs		Biologic DMARDs			
Conventional DMARDs	Targeted synthetic DMARDs (JAK inhibitors)	TNF inhibitors	IL-6R inhibitors	T cell co-stimulation inhibitors	B cell-depleting agents
Methotrexate leflunomide sulfasalazine hydroxy-chloroquine	Tofacitinib, baricitinib, filgotinib, upadacitinib, peficitinib	Etanercept, infliximab, adalimumab and biosimilars golimumab, certolizumab, pegol	Tocilizumab, sarilumab	Abatacept	Rituximab and biosimilars

IL-6R, interleukin-6 receptor; JAK, Janus kinase; TNF, tumor necrosis factor.

## Data Availability

Data sharing not applicable.

## Abbreviations

AA, adjuvant arthritis; ACPA, anti-citrullinated protein antibody; AIA, antigen-induced arthritis; Akt, protein kinase B; CAIA, collagen antibody-induced arthritis; CFA, complete Freund’s adjuvant; CIA, collagen-induced arthritis; COX-2, cyclooxygenase-2; CII, collagen type II; DMARD, disease-modifying anti-rheumatic drug; ERK, extracellular signal-regulated kinase; FLS, fibroblast-like synoviocyte; HLA, human leukocyte antigen; IFN-γ, interferon gamma; IκB, inhibitor of nuclear factor κB; IKK, IκB kinase; IL, interleukin; iNOS, inducible nitric oxide synthase; JAK, Janus kinase; JNK, c-Jun N-terminal kinase; LPS, lipopolysaccharide; MAPK, mitogen-activated protein kinase; MHC, major histocompatibility complex; MMP, matrix metalloproteinase; mTOR, mammalian target of rapamycin; MTX, methotrexate; NF-κB, nuclear factor kappa-B; NSAID, non-steroidal anti-inflammatory drug; PI3K, phosphoinositide 3-kinase; RA, rheumatoid arthritis; RANKL, receptor activator of nuclear factor kappa-B ligand; RF, rheumatoid factor; ROS, reactive oxygen species; STAT, signal transducer and activator of transcription; TGF-β, transforming growth factor beta; TLR, Toll-like receptor; TNF, tumor necrosis factor.
